# Targeting VGLUT2 in Mature Dopamine Neurons Decreases Mesoaccumbal Glutamatergic Transmission and Identifies a Role for Glutamate Co-release in Synaptic Plasticity by Increasing Baseline AMPA/NMDA Ratio

**DOI:** 10.3389/fncir.2018.00064

**Published:** 2018-08-29

**Authors:** Maria Papathanou, Meaghan Creed, Matthijs C. Dorst, Zisis Bimpisidis, Sylvie Dumas, Hanna Pettersson, Camilla Bellone, Gilad Silberberg, Christian Lüscher, Åsa Wallén-Mackenzie

**Affiliations:** ^1^Department of Organismal Biology, Uppsala University, Uppsala, Sweden; ^2^Department of Basic Neurosciences, University of Geneva, Geneva, Switzerland; ^3^Department of Neuroscience, Karolinska Institutet (KI), Solna, Sweden; ^4^Oramacell, Paris, France; ^5^Service of Neurology, Geneva University Hospital (HUG), Geneva, Switzerland

**Keywords:** cocaine, amphetamine, addiction, substance use disorder, ventral tegmental area (VTA), striatum, medium spiny neurons

## Abstract

Expression of the *Vglut2/Slc17a6* gene encoding the Vesicular glutamate transporter 2 (VGLUT2) in midbrain dopamine (DA) neurons enables these neurons to co-release glutamate in the nucleus accumbens (NAc), a feature of putative importance to drug addiction. For example, it has been shown that conditional deletion of *Vglut2* gene expression within developing DA neurons in mice causes altered locomotor sensitization to addictive drugs, such as amphetamine and cocaine, in adulthood. Alterations in DA neurotransmission in the mesoaccumbal pathway has been proposed to contribute to these behavioral alterations but the underlying molecular mechanism remains largely elusive. Repeated exposure to cocaine is known to cause lasting adaptations of excitatory synaptic transmission onto medium spiny neurons (MSNs) in the NAc, but the putative contribution of VGLUT2-mediated glutamate co-release from the mesoaccumbal projection has never been investigated. In this study, we implemented a tamoxifen-inducible Cre-LoxP strategy to selectively probe VGLUT2 in mature DA neurons of adult mice. Optogenetics-coupled patch clamp analysis in the NAc demonstrated a significant reduction of glutamatergic neurotransmission, whilst behavioral analysis revealed a normal locomotor sensitization to amphetamine and cocaine. When investigating if the reduced level of glutamate co-release from DA neurons caused a detectable post-synaptic effect on MSNs, patch clamp analysis identified an enhanced baseline AMPA/NMDA ratio in DA receptor subtype 1 (DRD1)-expressing accumbal MSNs which occluded the effect of cocaine on synaptic transmission. We conclude that VGLUT2 in mature DA neurons actively contributes to glutamatergic neurotransmission in the NAc, a finding which for the first time highlights VGLUT2-mediated glutamate co-release in the complex mechanisms of synaptic plasticity in drug addiction.

## Introduction

Drug addiction is a multifaceted neuropsychiatric disease characterized by a neurobiological interplay between the reinforcing effect of supraphysiological dopamine (DA) levels upon initial drug intake and the lasting alterations in glutamatergic synaptic strength upon repeated drug consumption (Volkow and Morales, 2015; Lüscher, 2016). Converging on medium spiny neurons (MSNs) in the nucleus accumbens (NAc) of the ventral striatum, the main DA input is derived from DA neurons located in the ventral tegmental area (VTA) of the midbrain while substantial glutamatergic innervation originates in numerous cortical and limbic sources including the prefrontal cortex, amygdala and hippocampus (Yager et al., 2015). In addition to forebrain-derived glutamatergic transmission within the NAc, a group of VTA DA neurons possess the capacity for glutamate release within this area. Based on their unique capacity to co-release glutamate and DA, these VTA neurons, often referred to as “dual-signaling,” “bi-lingual,” “combinatorial” or “co-releasing” neurons (El Mestikawy et al., 2011; Trudeau et al., 2014; Pupe and Wallén-Mackenzie, 2015; Morales and Margolis, 2017), might be of particular interest to drug addiction, but their role in VTA circuitry and behavior remains to be fully clarified.

Historically, pioneering electrophysiological experiments in cell culture and brain slices led to the first evidence that VTA DA neurons can release glutamate, which was detected as excitatory postsynaptic currents (EPSCs) (Sulzer et al., 1998; Bourque and Trudeau, 2000; Joyce and Rayport, 2000; Chuhma et al., 2004, 2009; Dal Bo et al., 2004; Lavin et al., 2005). Expression of the *Vglut2 (*aka *Slc17a6)* gene encoding the Vesicular glutamate transporter 2 (VGLUT2) in cultured VTA DA neurons subsequently provided the means for vesicular co-release of glutamate (Dal Bo et al., 2004). Histological gene expression analyses of *Vglut2* and *Tyrosine hydroxylase* (*Th*), encoding the rate-limiting enzyme of DA synthesis (TH), in adult rodents confirmed the presence of cells having both Vglut2 mRNA and TH immunoreactivity within a subset of cells in the medial VTA, intermingled with cells showing either *Th* or *Vglut2* gene expression only (Kawano et al., 2006; Yamaguchi et al., 2007, 2011, 2015; Nair-Roberts et al., 2008). In addition to mice and rats, the VGLUT2/TH combinatorial cell type has also been identified within the VTA of non-human primates and humans (Root et al., 2016). Vglut2 mRNA has been visualized by *in situ* hybridization throughout several developing brain regions at embryonal day (E) 12.5 in the mouse, and co-localizes already at this stage with TH immunoreactivity within midbrain DA neurons (Birgner et al., 2010; Nordenankar et al., 2014). RT-PCR experiments have shown that 25% of Th-positive DA neurons express the *Vglut2* gene at birth, while only 14% keeps this expression after 6 weeks (Mendez et al., 2008). In rats, even fewer *Vglut2*-expressing DA neurons have been reported at adulthood (Yamaguchi et al., 2007). Immunohistological co-labeling analysis of terminals in the NAc further proposed age as factor of importance for the ability for glutamate-DA co-release, as the extent of co-localization between VGLUT2 and TH proteins regressed with age and was not visible in adult rats (Bérubé-Carrière et al., 2009; Moss et al., 2011). Optogenetic stimulations carried out in DA transporter (DAT)-Cre transgenic mice expressing Channelrhodopsin-2 (ChR2) in the VTA, however, demonstrated the presence of DAT-Cre-dependent EPSCs in accumbal MSNs of adult mice, thus verifying mesoccumbal glutamate release from DA neurons located within the VTA (Stuber et al., 2010; Tecuapetla et al., 2010). Responses were blocked by the selective AMPA antagonist DNQX, thus verifying their glutamatergic nature. It was also established that the recorded glutamate was released directly from DA neurons, that it occurred independent of DA activity and that glutamate release was accompanied by DA release (Stuber et al., 2010; Tecuapetla et al., 2010). Recently, optogenetic stimulations were shown to cause glutamate and DA release from distinct sites and vesicles originating within the same mesoaccumbal axons, thereby supporting dual release (co-release) from one axon but from distinct axonal substructures (Zhang et al., 2015).

The lack of selective approaches in experimental animals has made it challenging to address the behavioral role of glutamate-DA co-release (Pupe and Wallén-Mackenzie, 2015; Morales and Margolis, 2017). However, implementing DAT-Cre-driven targeting of the *Vglut2* gene, generating *Vglut2^lx/lx;DAT-Cre^* conditional knockout (KO) mice, several studies have implicated glutamate-DA co-release in reward processing of relevance to addiction (Birgner et al., 2010; Hnasko et al., 2010; Alsiö et al., 2011; Fortin et al., 2012). For example, *Vglut2^lx/lx;DAT-Cre^* KO mice showed altered psychomotor activity in response to both acute and repeated administration of drugs of abuse, including amphetamine and cocaine (Birgner et al., 2010; Hnasko et al., 2010; Fortin et al., 2012). Glutamatergic EPSCs in accumbal MSNs were shown to be completely abolished in such KO mice (Hnasko et al., 2010; Stuber et al., 2010; Wang et al., 2017), thus verifying the importance of VGLUT2 for mesoaccumbal glutamate-DA co-release. Further, when *Vglut2^lx/lx;DAT-Cre^* KO mice were tested in the operant self-administration paradigm, striking differences were observed when compared to control mice (Alsiö et al., 2011). *Vglut2^lx/lx;DAT-Cre^* KO mice consumed more sugar than controls and had increased consumption of cocaine at a low dose. The results suggested that loss of VGLUT2 heightens the sensitivity to palatable food (sugar) and to addictive drugs (cocaine) (Alsiö et al., 2011).

Importantly, all gene targeting studies of mesoaccumbal glutamate-DA co-release performed so far suffer from the uncertainty that any observed phenotypes might depend on developmental circuitry adaptations, as the endogenous promoters of both *Vglut2* (Birgner et al., 2010; Nordenankar et al., 2014) and *Dat* (Ang, 2006) genes have embryonal onset. The proposed age-dependent decrease in *Vglut2* expression within the VTA toward adulthood alongside the developmental component of DAT-Cre-driven *Vglut2* gene targeting has made it impossible to dissociate the putative role of VGLUT2 in mature DA neurons from developmental compensatory adaptations. Further, on a molecular and circuitry level, while ablation of VGLUT2 has been shown to affect DA release in the NAc (Hnasko et al., 2010; Alsiö et al., 2011), the physiological role of the co-released glutamate in mesoaccumbal neurotransmission has remained unexplored.

To unambiguously pinpoint the role of *Vglut2* gene expression in mature DAT-Cre neurons, this study implemented a tamoxifen-inducible DAT-Cre transgene (DAT-CreERT2; Engblom et al., 2008) to control temporal aspects of recombination. We found that ablation of *Vglut2* gene expression in mature DA neurons significantly decreased excitatory post-synaptic currents (EPSCs) in the NAc. Behaviorally, and in stark contrast to developmental VGLUT2 targeting, DAT-CreERT2-induced VGLUT2 targeting in adulthood did not disturb psychostimulant-induced locomotion. By addressing synaptic plasticity in the NAc, we found that DA receptor 1 (DRD1)-expressing MSNs showed normal rectification index (RI) but increased baseline AMPA/NMDA ratio, which cocaine did not increase further. This study thereby identifies a role for VGLUT2-mediated glutamate co-release from DAT-Cre-positive neurons in synaptic plasticity of putative relevance to drug addiction.

## Materials and Methods

### Animal Housing

Animals were housed on a standard 12 h sleep/wake cycle (7:00 A.M. lights on, 7:00 P.M. lights off). Mice were provided with food and water *ad libitum* unless stated otherwise and housed according to Swedish legislation (Animal Welfare Act SFS 1998:56) and European Union legislation (Convention ETS 123 and Directive 2010/63/EU). All experiments were conducted with permission from the local Animal Ethical Committees (Uppsala University, UU/Karolinska Institutet, KI) and by the Institutional Animal Care and Use Committee (University of Geneva, UG), respectively. For glutamate recordings upon optogenetic stimulation, mice were transferred from UU to KI, Stockholm. For cocaine sensitization and electrophysiological recordings, mice were transferred from UU to UG.

### Generation and Genotyping of Transgenic Mice

Genotyping of transgenic mice was performed by PCR analysis (Supplementary Table S1). The following Cre-drivers were implemented in the study: (i) DAT-Cre transgenic mouse line with embryonal onset of the transgene (Ekstrand et al., 2007), here abbreviated eDAT-Cre; and (ii) Tamoxifen-inducible DAT-CreERT2 mice (Engblom et al., 2008), here abbreviated txDAT-Cre. Mice of the eDAT-Cre and txDAT-Cre transgenic lines were bred with the Vglut2^lx^ conditional KO mouse line in which exons 4–6 are surrounded by LoxP sites to enable Cre-driven *Vglut2* gene targeting (Wallén-Mackenzie et al., 2006). DAT-Cre driven recombination of the floxed *Vglut2* gene directs *Vglut2* gene targeting to DA neurons either during development (eDAT-Cre-driver: *Vglut2^eDAT-Cre^* transgenic line) or in mature DA neurons upon tamoxifen treatment (txDAT-Cre-driver: *Vglut2^txDAT-Cre^* transgenic line), respectively. Offspring within these lines were used for behavioral and electrophysiological experiments. *Vglut2^eDAT-Cre^* and *Vglut2^txDAT-Cre^* transgenic lines were also bred to the floxed tdTom reporter line (B6;129S6-*Gt (ROSA) 26Sor^tm14(CAG-tdTomato)Hze^*/J (Jackson Laboratory) and to the DRD1a-EGFP reporter line (Tg(Drd1-EGFP)X60Gsat/Mmmh; Gensat) for fluorescent visualization of Cre-driven activity and of neurons expressing the DA receptor subtype 1 (DRD1), respectively.

### Tamoxifen Administration

All experimental mice containing the txDAT-Cre allele were treated with tamoxifen to induce recombination (Sigma, T-5648). Tamoxifen was dissolved in sunflower oil and ethanol (9:1) to a final concentration of 20 mg/ml. Animals (8–9 week old) were intraperitoneally injected with 2 mg of tamoxifen once daily for five consecutive days. Animals undergoing stereotaxic injections were treated with their last tamoxifen injection 10 days prior to surgery. All behavioral and electrophysiological experiments commenced no earlier than 1 week after last tamoxifen injection.

### RNA Extraction and Nested PCR

Brain tissue from tamoxifen-treated *Vglut2^lx/lx;txDAT-Cre-tg^* and *Vglut2^lx/lx;txDAT-Cre-wt^* mice was collected 1 week after tamoxifen treatment, snap-frozen in −30°/−35°C isopentane and stored at −80°C until further use. The VTA was dissected out using the 2 mm brain punch and a brain matrice (Agnthos, Sweden) at −20°C. The RNA from the dissected VTA was extracted using the Qiagen RNeasy Plus Mini Kit according to manufacturer’s guidelines. Ten nanogram of total RNA, Oligo (dT)_20_ primer and the ThermoScriptRT kit (Invitrogen) was used for cDNA synthesis. PCR was performed using Phusion High-Fidelity DNA Polymerase kit (ThermoFisher Scientific) with a total volume of 20 μl. The first PCR reaction (PCR1) contained 1.5 μl of cDNA and the second PCR (PCR2) contained 1 μl of PCR1 (Supplementary Table S2 for primer sequences and thermal conditions). Five microliter of the final product of PCR2 was subjected to agarose gel electrophoresis using 2.8% agarose.

### Stereotaxic Injection of Optogenetic Virus

*Vglut2^lx/lx;txDAT-Cre-tg^*, *Vglut2^wt/lx;txDAT-Cre-tg^* and *Vglut2^wt/wt;txDAT-Cre-tg^* littermate mice (>8 weeks; >20 g) were anesthetized with isoflurane and stereotaxically injected unilaterally into the VTA with 300 nl of AAV5-EF1a-DIO-ChR2(H134)-eYFP (titer 5.6 × 10^12^ vg/ml; UNC Gene Therapy, Chapel Hill, NC, USA) at a flow-rate of 100 nl per min at the following coordinates from Bregma: anterior-posterior −3.45, medial-lateral −0.2 and dorsal-ventral −4.4 according to Paxinos and Franklin (2012). Topical analgesic, Marcaine (1.5 mg/kg; AstraZeneca) was applied during surgery and Caprofen (5 mg/kg; Norocarp) was given subcutaneously pre- and post-surgery. All animals were allowed to recover for at least 2 weeks prior to electrophysiological recordings. Mice received tamoxifen treatment prior to virus injections as described above.

### Histological Analysis

#### Immunohistochemistry

Deeply anesthetized mice were transcardially perfused with body-temperature phosphate-buffered saline (PBS) followed by ice-cold 4% formaldehyde. Brains were dissected and post-fixed overnight. The brains were then cryo-protected with 30% sucrose and cut using a cryostat at 60 μm slice thickness. Free-floating sections were processed for immunofluorescence according to standard protocols (Primary Antibodies: rabbit TH 1:1,000 #ab172, Millipore; chicken GFP 1:1,000 #ab13970, Abcam; Secondary antibodies: Donkey Anti-Rabbit Alexa Fluor 488 1:500; Donkey-Anti-Chicken Alexa Fluor 488 1:500; Donkey Anti-Rabbit Cy3 1:500). The signal of tdTom was detected by endogenous fluorescence without any additional use of an antibody. Images were captured using Mirax MIDI scanner or a Zeiss Confocal (LSM 700, 20× magnification) and analyzed using PanoramicViewer or Zen software. Manual quantification of TH-positive neurons in the VTA and SNc was performed on three sections along the anterio-posterior axis for each animal (*Vglut2^lx/lx;txDAT-Cre-wt^*, *n* = 3; *Vglut2^lx/lx;txDAT-Cre-tg^*, *n* = 3). Analysis was done by two-way ANOVA with Sidak *post hoc* test. Brain slices (250 μm) used for patch clamp recordings were fixed overnight in Lana’s fixative, for detection of neurobiotin and GFP. The sections were subsequently washed overnight in 0.01 M phosphate buffered saline (PBS), followed by another overnight incubation with Cy5-conjugated streptavidin (1:1,000) in 0.1 M PB containing 0.3% Triton X-100. They were then washed and incubated in PBS with DAPI (1:1,000) for 30 min and washed again before mounting with Fluoromount Aqueous mounting medium (Sigma, USA) and analyzed. GFP was detected by endogenous fluorescence without any additional enhancement of signal.

#### *In situ* Hybridization

The brains of anesthetized mice were extracted and snap-frozen by rapidly immersing the tissue in ice-cold isopentane (−25°/−30°C for embryo E14.5, postnatal day 3; −30°/−35°C for adult). Colorimetric and double fluorescent *in situ* hybridization analyses were performed as previously described (Viereckel et al., 2016; Riboprobes corresponding to: slc17a6 NM_080853.3, sequence: 2315–3244; tdTomato, sequence: 84–653, slc6a3 NM_012694.2 sequence 1015–1938, th NM_012740.3, sequence 456–1453). All slides were scanned at 20× magnification on a NanoZoomer *2.0-HT*. The Ndp2.view software (Hamamatsu) was employed for viewing the images and manual counting was performed for quantification (Post-natal (P) day 3, *n* = 3; Adult, *n* = 3).

### Behavioral Analysis

All behavioral experiments took place during the light-cycle (8:00 AM to 6:00 PM). All animals were adult (≥8 weeks) and habituated to handling for several days prior to behavioral assessment. Both male and female mice were used throughout the study.

#### Amphetamine-Induced Behavioral Sensitization

Locomotor behavior of *Vglut2^lx/lx;eDAT-Cre-tg^*, *Vglut2^lx/lx;eDAT-Cre-wt^*, *Vglut2^lx/lx;txDAT-Cre-tg^* and *Vglut2^lx/lx;txDAT-Cre-wt^* mice was recorded upon amphetamine injections (3 mg/kg i.p) using the Ethovision XT 13.0 software. *Vglut2^lx/lx;txDAT-Cre-tg^* and *Vglut2^lx/lx;txDAT-Cre-wt^* mice received tamoxifen treatment prior to the onset of the experiment. Amphetamine-induced locomotor activity was analyzed in open-field activity boxes (Makrolon^®^ polycarbonate cages, 15 × 26 × 40 cm) containing 1.5 cm bedding and a transparent plexiglas lid. The mice were allowed to habituate to the boxes and surrounding environment for 30 min prior to drug administration. Locomotor activity was measured for 90 min consisting of (30 min baseline and 60 min drug-induced activity). The experimental setup consisted of saline treatment on day 1, followed by four consecutive days of amphetamine injections (days 2–5) and a subsequent acute amphetamine challenge 2 weeks later (day 19). Mice were then left to recover for 1 week and on day 26 received another saline injection followed by four subsequent amphetamine injections on days 27–30. After 1 week the mice received an acute amphetamine challenge (day 36), and a final amphetamine challenge 1 week later (day 44). Statistical analysis was performed with GraphPad Prism 7 using 2-way repeated measures ANOVA with Sidak *post hoc* test using genotype and time as variables (*Vglut2^lx/lx;eDAT-Cre-tg^*, *n* = 3; *Vglut2^lx/lx;eDAT-Cre-wt^*, *n* = 3; *Vglut2^lx/lx;txDAT-Cre-tg^*, *n* = 11 and *Vglut2^lx/lx;txDAT-Cre-wt^*, *n* = 7. Males *n* = 15, Females *n* = 9).

### Electrophysiological Recordings

#### Patch Clamp Recordings Upon Optogenetic Stimulation

Post-synaptic currents upon optogenetic stimulation were analyzed by patch clamp recordings in brain slices from tamoxifen-treated *Vglut2^lx/lx;txDAT-Cre-tg^*, *Vglut2^wt/lx;txDAT-Cre-tg^* and *Vglut2^wt/wt;txDAT-Cre-tg^* mice stereotaxically injected unilaterally into the VTA with AAV5-EF1a-DIO-ChR2(H134)-eYFP as described above. Mice were anesthetized using isoflurane and dissected brains immersed in ice cold cutting solution containing (in mM): KCl 2.5, NaH_2_PO_4_ · H_2_O 1.25, CaCl_2_ · 2H_2_O 0.5, MgCl_2_ · 6H_2_O 7.5, Glucose 10, NaHCO_3_ 25, and Sucrose 205. Coronal sections 250 μm thick were cut on a VT1200S Vibratome (Leica, Japan) and recovered for 30 min in 35°C artificial cerebrospinal fluid (ACSF) containing (in mM): NaCl 125, KCl 2.5, MgCl_2_ · 6H_2_O 1, NaH_2_PO_4_ · H_2_O 1.25, CaCl_2_ · 2H_2_O 2, Glucose 25, and NaHCO_3_ 25. Slices were maintained at room temperature until recording at 35°C. Solutions were continuously oxygenated with carbogen (95% O_2_, 5% CO_2_) throughout the procedure. Borosilicate glass pipettes were pulled on a P1000 micropipette puller (Sutter Instruments, Novato, CA, USA) to a resistance of 4–6 MΩ and filled with intracellular solution containing (in mM): CsCl 10, CsMeSO_3_ 110, HEPES 10, Na_2_-Phosphocreatine 10, ATP-Mg 4, GTP-Na 0.3, TEA-Cl 10 and QX-314 Cl 1. For staining, 0.3% neurobiotin was added to the intracellular solution and detected as described above (“Immunohistochemistry” section). Neurons were identified using Infrared-Differential Interference Contrast (IR-DIC) imaging on a BX51WI (Olympus, Japan) upright microscope equipped with a 40× long-working-distance immersion objective. Neurons were selected by proximity to afferent fibers. Once a whole-cell patch was achieved, light evoked responses were recorded in V_H_ = −70 mV voltage-clamp on a MultiClamp 700B (Molecular Devices, Sunnyvale, CA, USA), digitized at 10 KHz on a ITC-18 (HEKA, Houston, TX, USA) and acquired with Igor Pro 6.3 (Wavemetrics, Tigard, OR, USA). Fibers were stimulated through an ocular-mounted blue LED producing 6.4 mW/mm^2^ light under the objective, controlled through an SLA-1200-2 LED driver (Mightex, Pleasanton, CA, USA). When EPSCs could reliably be evoked, 5 μm NBQX was bath-applied and light-evoked EPSCs were again recorded. Peak amplitude was determined by subtracting the mean current in the 100 ms preceding light stimulation from the maximum deviation achieved in the 100 ms following light onset. Data were analyzed using unpaired Mann-Whitney test (*Vglut2^wt/lx;txDAT-Cre-tg^* and *Vglut2^wt/wt;txDAT-Cre-tg^* animals were pooled together as controls (txCtrl) as there were no significant difference between these groups; txCtrl, cells *n* = 24, 3 mice and *Vglut2^lx/lx;txDAT-Cre-tg^* (txKO), cells *n* = 24, 3 mice. Males *n* = 4; Females *n* = 2).

#### Patch Clamp Recording Upon Cocaine-Induced Behavioral Sensitization

Locomotor behavior of *Vglut2^lx/lx;eDAT-Cre-tg^*, *Vglut2^lx/lx;eDAT-Cre-wt^*, *Vglut2^lx/lx;txDAT-Cre-tg^* and *Vglut2^lx/lx;txDAT-Cre-wt^* mice carrying the DRD1-EGFP transgene (see “Generation and Genotyping of Transgenic Mice” section above) was recorded upon cocaine injections whereupon post-synaptic currents were recorded in patch clamp electrophysiology. *Vglut2^lx/lx;txDAT-Cre-tg^* and *Vglut2^lx/lx;txDAT-Cre-wt^* mice received tamoxifen treatment prior to the experimental start. Both male and female mice were used. Cocaine-induced locomotor activity was measured as the distance traveled in a circular corridor (outer/inner diameter, 30/10 cm; video tracking system, Anymaze, Stoeling). After 3 days of habituation, locomotion was recorded following five consecutive days of saline or cocaine (20 mg/kg, i.p.; Sigma) administration. Behavioral recording took place for 80 min, which consisted of 20 min habituation and 60 min of saline or cocaine-induced locomotion (saline: *Vglut2^lx/lx;eDAT-Cre-wt^*, *n* = 3; *Vglut2^lx/lx;eDAT-Cre-tg^*, *n* = 2, cocaine: *Vglut2^lx/lx;eDAT-Cre-wt^*, *n* = 5; *Vglut2^lx/lx;eDAT-Cre-tg^*, *n* = 5, saline: *Vglut2^lx/lx;txDAT-Cre-wt^*, *n* = 4; *Vglut2^lx/lx;txDAT-Cre-tg^*, *n* = 4, cocaine: *Vglut2^lx/lx;txDAT-Cre-wt^*, *n* = 9; *Vglut2^lx/lx;txDAT-Cre-tg^*, *n* = 8).

After 10 days, coronal mouse brain slices were prepared in cooled artificial cerebrospinal fluid containing (in mM): 119 NaCl, 2.5 KCl, 1.3 MgCl, 2.5 CaCl_2_, 1.0 Na_2_HPO_4_, 26.2 NaHCO_3_ and 11 glucose, bubbled with 95% O_2_ and 5% CO_2_. Slices were kept at 32–34°C in a recording chamber superfused with 2.5 ml/min artificial cerebrospinal fluid. Visualized whole-cell voltage-clamp recording techniques were used to measure holding and synaptic responses of DRD1-MSNs of the NAc shell, identified by the presence of the DRD1a-EGFP reporter. Holding potential was maintained at −70 mV, and access resistance was monitored by a depolarizing step of −14 mV each sweep, every 10 s. The liquid junction potential was small (−3 mV); therefore, traces were not corrected. Experiments were discarded if the access resistance varied by more than 20%. Currents were amplified, filtered at 5 kHz and digitized at 20 kHz. All experiments were performed in the presence of picrotoxin (100 μm) to isolate excitatory transmission. The internal solution contained (in mM) 130 CsCl, 4 NaCl, 5 creatine phosphate, 2 MgCl_2_, 2 Na_2_ATP, 0.6 Na_3_GTP, 1.1 EGTA, 5 HEPES and 0.1 mm spermine. Synaptic currents were electrically evoked by stimuli (50–100 μs) at 0.1 Hz through bipolar stainless steel electrode placed onto the tissue. To isolate AMPAR-evoked EPSCs, the NMDA antagonist D-AP5 (50 μm) was bath applied. The NMDAR component was calculated as the difference between the EPSCs measured in the absence and presence of D-AP5. The AMPAR/NMDAR ratio was calculated by dividing the peak amplitudes. The RI of AMPAR-mediated currents was calculated as the ratio of the chord conductance calculated at −70 mV, divided by chord conductance at +40 mV. In sample traces, stimulation artifacts were removed. RI saline: *Vglut2^lx/lx;eDAT-Cre-tg^*, *n* = 5, *Vglut2^lx/lx;eDAT-Cre-wt^*, *n* = 4; cocaine *Vglut2^lx/lx;eDAT-Cre-tg^*, *n* = 28, *Vglut2^lx/lx;eDAT-Cre-wt^*, *n* = 12; AMPA/NMDA ratio saline: *Vglut2^lx/lx;eDAT-Cre-tg^*, *n* = 5, *Vglut2^lx/lx;eDAT-Cre-wt^*, *n* = 7; cocaine: *Vglut2^lx/lx;eDAT-Cre-tg^*, *n* = 33, *Vglut2^lx/lx;eDAT-Cre-wt^*, *n* = 15. RI saline: *Vglut2^lx/lx;txDAT-Cre-tg^*, *n* = 6, *Vglut2^lx/lx;txDAT-Cre-wt^*, *n* = 6; cocaine *Vglut2^lx/lx;txDAT-Cre-tg^*, *n* = 7, *Vglut2^lx/lx;txDAT-Cre-wt^*, *n* = 12; AMPA/NMDA ratio saline: *Vglut2^lx/lx;txDAT-Cre-tg^*, *n* = 9, *Vglut2^lx/lx;txDAT-Cre-wt^*, *n* = 9; cocaine: *Vglut2^lx/lx;txDAT-Cre-tg^*, *n* = 8, *Vglut2^lx/lx;txDAT-Cre-wt^*, *n* = 13. Data were analyzed with two-way repeated measures ANOVA and Tukey’s *post hoc* test (behavior) or two-way ANOVA and Sidak *post hoc* (electrophysiological recordings). All mice expressed the DRD1-EGFP transgene.

## Results

### *Vglut2* Gene Highly Expressed Throughout Mouse Midbrain With Restricted Expression Within the Dopaminergic Area

**Figure 1:**
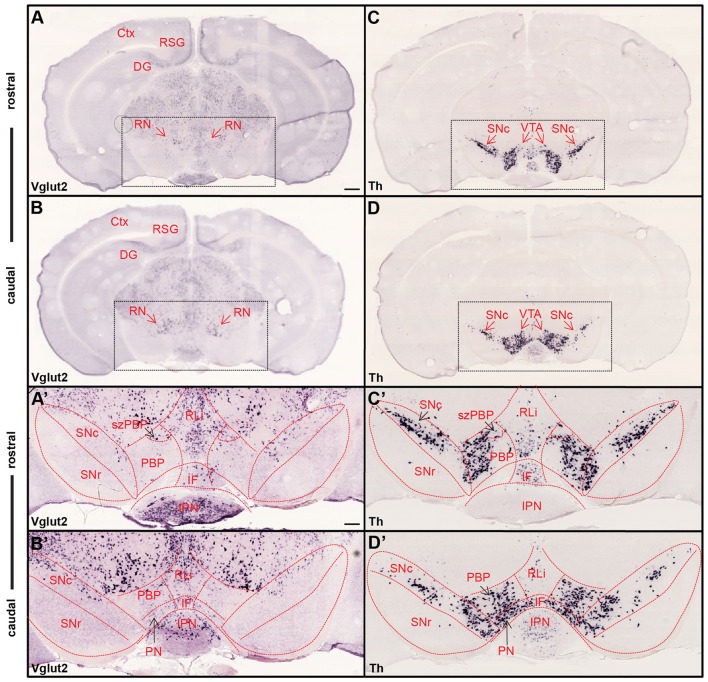
Ample Vglut2 mRNA-positive cells throughout dorsal and ventral midbrain with more sparse expression within the dopaminergic area. Colorimetric *in situ* hybridization showing overview of Vglut2 **(A,B)** and Th **(C,D)** mRNA in midbrain section of wildtype adult mouse at two rostro-caudal levels. **(A,B)** Vglut2 mRNA is abundant throughout the midbrain with strong signal in e.g., the red nucleus (RN), RSG and dentate gyrus and weaker signal in the ventral tegmental area (VTA) and SNc areas. **(C,D)** Th mRNA is selectively localized in dopaminergic neurons of the VTA and SNc and its mRNA signal implemented to visualize these areas. Dotted square around the VTA and SNc (scale bar 500 μm) presented as closeups in (**A′–D′**; scale bar 200 μm). **(C′,D′)** SNc, SNr and subregions of VTA outlined in Th closeups and superimposed on Vglut2 closeups **(A′,B′)**. **(C′,D′)** Th mRNA was strongly localized in the SNc and within the parabrachial pigmented area (PBP) and paranigral nucleus (PN) of the VTA with weaker signal in the RLi and caudal IF. **(A′,B′)** Within the VTA, Vglut2 mRNA was detected in the PBP, PN, RLi and IF as well as within the medially located szPBP while no Vglut2 mRNA was detected in the GABAergic SNr area. Abbreviations: Ctx, Cortex; DG, Dentate gyrus; IF, interfascicular nucleus; IPN, interpeducular nucleus; PBP, parabrachial pigmented area; PN, paranigral nuclei; RLi rostral linear nucleus; RN, Red nucleus; RSG, Retrosplenial granular cortex; SNc, Substantia nigra *pars compacta*; SNr, Substantia nigra *pars reticulata*; szPBP, subzone of the parabrachial pigmented area; VTA, Ventral tegmental area.

The presence of VGLUT2 molecules enables neurons to package the essential amino acid glutamate into presynaptic vesicles for fast synaptic neurotransmission upon depolarization (Fremeau et al., 2001; Herzog et al., 2001; Takamori et al., 2001; Varoqui et al., 2002). While this protein is localized in the presynaptic axonal terminals within projection target areas, Vglut2 mRNA locates to the cell soma. Thus, VGLUT2 immunohistochemistry will detect VGLUT2 molecules in the presynaptic terminal and Vglut2 mRNA-selective *in situ* hybridization will visualize the cell body from which these axons originate. By implementing *in situ* hybridization in adult mouse midbrain, we first confirmed previous findings in rat that Vglut2 mRNA is abundant throughout the midbrain (Kawano et al., 2006; Figures 1A,B). Using a probe detecting Th mRNA, the VTA and substantia nigra *pars compacta* (SNc) area containing midbrain DA neurons, were visualized in adjacent sections (Figures 1C,D). While DA neurons in the midbrain are confined within the VTA/SNc area, *Vglut2* gene expression was sparse within this area compared to its expression throughout the other areas of the midbrain, for example the red nucleus (RN) located immediately dorsal of the VTA. Th mRNA was used to illustrate the outline of the subareas of the VTA and the SNc (Figure 1C′,D′) which was subsequently superimposed on the images of Vglut2 mRNA (Figure 1A′,B′). Previous analyses of mouse and rat (Kawano et al., 2006; Yamaguchi et al., 2011, 2015) have shown that Vglut2 mRNA-positive cells are scattered throughout the mouse VTA and SNc with more frequent appearance medially than laterally and Vglut2/Th co-localization in the VTA but not in the SNc. Within the VTA, Vglut2 mRNA was detected in the parabrachial pigmented area (PBP) and paranigral nucleus (PN) as well as the medially located nuclei, rostral linear nucleus (RLi) and interfascicular (IF). As expected, no Vglut2 mRNA was detected in the GABAergic SNr area. We could show that within the VTA, highest density of Vglut2 mRNA is found in the RLi. Vglut2 mRNA was also prominent within the medially located subzone of the PBP (szPBP; described in Viereckel et al., 2016), but similar as RLi, less Th mRNA is found in this area (Figure 1A′,B′). These histological mRNA analyses confirmed previous findings of scattered Vglut2 mRNA in the VTA/SNc of the adult mouse midbrain. To address the spatio-temporal distribution of glutamate-DA co-releasing neurons in more detail, we next turned to double-labeling experiments.

### Number of *Vglut2/Dat* Co-expressing VTA Neurons Highest Around Birth and Reduced in Adulthood

**Figure 2:**
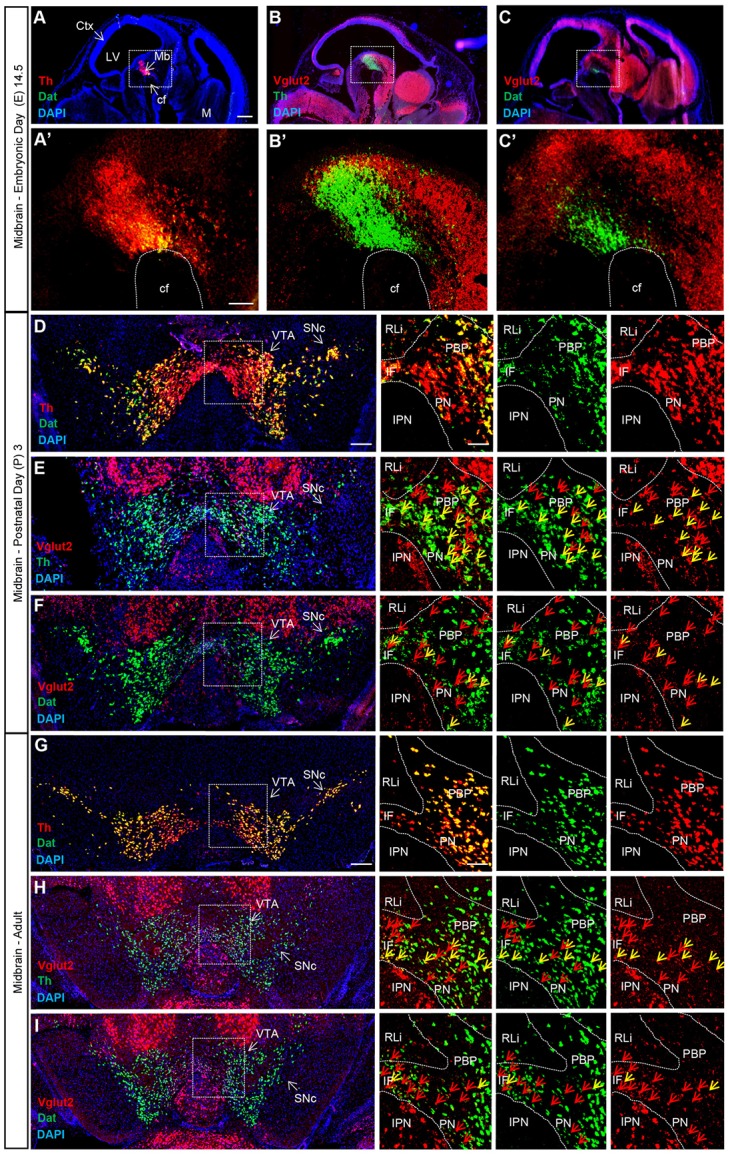
Vglut2, Th and Dat mRNA co-localization within certain VTA dopamine (DA) neurons is sparse at E14.5, peaks around birth and is subsequently down-regulated in adulthood. Double fluorescent *in situ* hybridization for Th (red), Dat (green) and Vglut2 (red) mRNA, respectively, on wildtype mouse midbrain sections. **(A–C)** Sagittal sections of E14.5 embryo. Dotted square around the area of developing midbrain DA neurons **(A–C)** with close-ups in **(A′–C′)**. **(A)** Th and Dat mRNA show co-localization (yellow) in the ventral midbrain (scale bar 500 μm). **(A′)** higher magnification of insets (scale bar 100 μm); **(B,B′)** Th and Vglut2 mRNA expression in the midbrain. **(C,C′)** Dat and Vglut2 mRNA show sparse detection in the midbrain. **(D–F)** Coronal sections of ventral midbrain in pups of postnatal day (P) 3. **(D)** Th and Dat show ample co-localization (yellow) in the lateral VTA and SNc (scale bar 250 μm, inset 100 μm). **(E)** Th and Vglut2 mRNA and **(F)** Dat and Vglut2 mRNA prominently co-localize (yellow) at this age in the IF, PBP and PN areas (arrows) but not in the RLi of the VTA. **(G–I)** Coronal sections of the adult midbrain (10 weeks; scale bar 250 μm, inset 100 μm). **(G)** Th and Dat mRNA co-localization (yellow) remains strong; whilst the level of co-localization between **(H)** Th and Vglut2 and **(I)** Dat and Vglut2 mRNAs is lower than at P3 (arrows). Yellow arrows show co-localization green (Dat) and red (Vglut2) channel, red arrows show red (Vglut2) channel (Postnatal Day (P) 3 *n* = 3; adult *n* = 3). Abbreviations: cf, cephalic flexture; Ctx, cortex; IF, interfascicular nucleus; IPN, interpeducular nucleus; LV, lateral ventricle; M, medulla; Mb, midbrain; PBP, parabrachial pigmented area; PN, paranigral nuclei; RLi rostral linear nucleus; SNc, Substantia nigra *pars compacta*; VTA, Ventral tegmental area. See Supplementary Figure S1 for low-magnification images of entire sections at P3 and adult as well as quantification Vglut2/Dat mRNA co-localization.

Combinatorial neurons expressing both *Vglut2* and *Th* genes comprise a minority of the total cells in the adult VTA expressing either *Th* or *Vglut2* genes (Yamaguchi et al., 2011, 2013; Morales and Root, 2014). In previous studies, we have shown that Vglut2 mRNA can be detected within TH-positive DA neurons of the developing midbrain already at E12.5 in the mouse embryo (Birgner et al., 2010; Nordenankar et al., 2014), while other studies have demonstrated an age-dependent decrease in Vglut2 mRNA in TH neurons from birth into adulthood (Yamaguchi et al., 2007; Mendez et al., 2008). TH is a crucial enzyme in the biosynthesis pathway for DA and hence, all DA neurons express its gene by definition. In contrast, medial VTA DA neurons have less DAT, the extracellular DA transporter, than DA neurons in the lateral VTA and SNc in the adult midbrain (Lammel et al., 2008; Li et al., 2013; Viereckel et al., 2016). To address and compare the temporal and spatial distribution of Vglut2 mRNA in Th- and Dat-positive neurons, we implemented double fluorescent *in situ* hybridization to co-localize Vglut2 mRNA with Th and Dat mRNA, respectively. Th mRNA has been described around E11 and Dat mRNA has been reported to appear around E13 (Ang, 2006). We addressed E14.5 as youngest stage and prepared multiple sections throughout the midbrain at E14.5, post-natal day 3 (P3) and in adulthood (10 weeks). At E14.5, Th mRNA was readily detected in the ventral midbrain where Dat mRNA co-localized with Th ventrally (Figure 2A,A′). Sparse co-localization was detected between Th and Vglut2 mRNA (Figure 2B,B′) and between Dat and Vglut2 mRNA at this embryonal stage (Figure 2C,C′).

At P3, Th and Dat mRNA showed ample co-localization in the lateral VTA and SNc, while Th mRNA was stronger than Dat mRNA in the medial VTA, confirming previous finding using the same method (Viereckel et al., 2016; Figure 2D). Vglut2 mRNA showed prominent co-localization with both Th and Dat mRNA at this stage, however, the density of Vglut2/Th double-positive cells was higher than Vglut2/Dat double-positive cells (Figures 2E,F and Supplementary Figure S1A). Subareas within the VTA showed different amount of co-localization at P3. Primarily the PBP, but also the PN and IF, showed co-localization of Vglut2 with Th and Dat, while the more dorsally located RLi, which shows the highest level of Vglut2 mRNA, was almost devoid of co-localization with either Th or Dat mRNA (Figures 2E,F).

In the adult mouse, Th and Dat mRNA showed substantial overlap and also at this stage, the overlap was stronger in the lateral VTA and SNc than in the medial VTA where Th was stronger than Dat (Figure 2G). In contrast, the level of Vglut2/Th and Vglut2/Dat double-positive cells was low in all VTA subareas (Figures 2H,I). Vglut2 mRNA, more medially than laterally located, showed more overlap with Th than with Dat also at this stage (Figures 2H,I and Supplementary Figure S1B). Quantification showed 128 (±10) vs. 24 (±5) Vglut2/Dat mRNA-double-positive cells in P3 vs. adult VTA (Supplementary Figure S1C). Together, these histological results show that *Vglut2* gene expression in Dat-positive DA neurons of the VTA is highest around birth with temporal down-regulation towards adulthood. Areas with highest level of Vglut2 mRNA (RLi and szPBP) show only low levels of Dat mRNA and are sparse also in Th mRNA. The presence of Vglut2 mRNA in mature Dat mRNA-positive neurons, if yet very few, might be sufficient to account for previously observed glutamate co-release in the mesoaccumbal pathway. We decided to address the role of this sparse presence of VGLUT2 within DAT neurons of the adult mouse by implementing a temporally controlled targeting strategy.

### Tamoxifen-Induced Targeting of VGLUT2 in DA Neurons of Adult Mice Causes No Gross Morphological Alteration of Midbrain DA System

To specifically dissociate the role of VGLUT2 in mature DA neurons, we next employed the DAT-Cre-ERT2 transgenic mouse line in which the DAT-Cre transgene is temporally restricted (Engblom et al., 2008). By coupling of the Cre transgene to a mutated form of the estrogen nuclear hormone receptor (ERT2), the CreERT2 recombinase in these mice has been placed under control of the DAT promoter but the protein only translocates into the nucleus upon binding to the synthetic ligand tamoxifen. Recombination of any floxed alleles by the CreERT2 recombinase is thereby controlled by the time of injection of tamoxifen, which is delivered to the mouse by intraperitoneal injection (Engblom et al., 2008).

**Figure 3:**
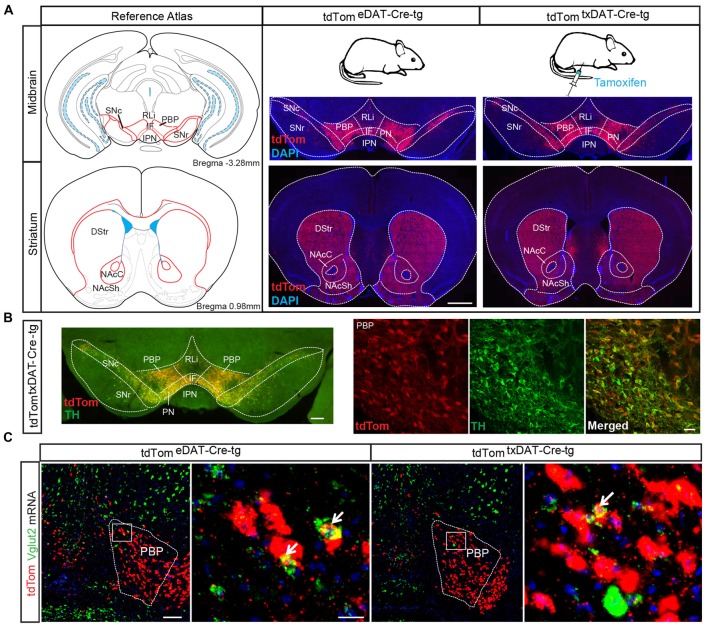
Validation of tamoxifen-inducible DAT-Cre-mediated targeting via tdTom reporter. **(A)** tdTom-immunohistochemical analysis of midbrain and striatal sections from *tdTom^eDAT-Cre-tg^* and *tdTom^txDAT-Cre-tg^* mice show similar extent of labeling in both mouse lines, verifying a similar recombination efficiency of LoxP sites (scale bar 2 mm). **(B)** tdTom-positive labeling (red) within the VTA and SNc co-localizes with tyrosine hydroxylase (TH; green) immuno-labeling verifying selectivity to DA neurons (scale bar 250 μm and 50 μm). **(C)** Low and high magnification of fluorescent *in situ* hybridization for Vglut2 (green) and tdTom (red) mRNA in *tdTom^eDAT-Cre-tg^* and *tdTom^txDAT-Cre-tg^* mice showing co-localization in the PBP, albeit at low level (scale bar 200 μm; inset 25 μm). Abbreviations: DStr, Dorsal striatum; IF, interfascicular nucleus; IPN, interpeducular nucleus; NAcC, Nucleus accumbens core; NAcSh, Nucleus accumbens shell; PBP, parabrachial pigmented area; PN, paranigral nuclei; RLi, rostral linear nucleus; SN, Substantia nigra; SNc, Substantia nigra *pars compacta*; SNr, Substantia nigra *pars reticulata*; VTA, Ventral tegmental area; tdTom, tdTomato.

Since we have implemented and compared two different Cre-driver lines under control of the DAT promoter in this study, we will use the following abbreviations from now onwards: The embryonically active DAT-Cre (Ekstrand et al., 2007) has been abbreviated as eDAT-Cre while the tamoxifen-inducible DAT-Cre-ERT2 (Engblom et al., 2008) has been abbreviated as txDAT-Cre (see also “Materials and Methods” section). Throughout the study, txDAT-Cre mice were injected with tamoxifen at 8–9 weeks of age. First, to verify and compare spatial and temporal specificity of the Cre drivers, eDAT-Cre and txDAT-Cre transgenic mice were both bred with tdTom reporter mice. Offspring, *tdTom^eDAT-Cre-tg^* and *tdTom^txDAT-Cre-tg^* mice, were analyzed with histological methods. When comparing *tdTom^eDAT-Cre-tg^* mice with the tamoxifen-induced *tdTom^txDAT-Cre-tg^* mice, similar expression patterns of tdTom reporter gene expression were seen in midbrain DA areas VTA (including subareas PBP, RLi, IF, PN) and SNc as well as in striatal target regions, the dorsal striatum (DStr) and the NAc (both core and shell subareas, NAcC and NAcSh) of the ventral striatum (Figure 3A). The eDAT-Cre and txDAT-Cre drivers thus seem to have similar efficiency in mediating recombination of LoxP sites. As expected, tdTom overlapped well with TH immunoreactivity (Figure 3B). A subset of tdTom cells within the VTA, mainly within the PBP subarea, showed co-localization between tdTom and Vglut2 mRNA (Figure 3C).

**Figure 4:**
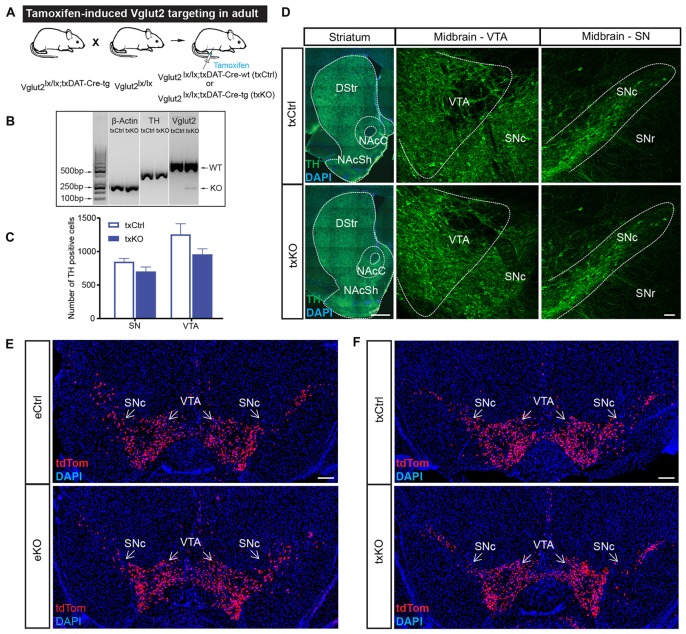
Confirmation of tamoxifen-induced targeting of the *Vglut2* gene using tamoxifen-inducible DAT-Cre transgene and validation of intact midbrain DA system. **(A)** Schematic illustration of breeding strategy to generate *Vglut2^lx/lx;txDAT-Cre-wt^* (txCtrl) and *Vglut2^lx/lx;txDAT-Cre-wt^* (txKO). **(B)** Confirmation of *Vglut2* gene targeting using the tamoxifen-inducible DAT-CReERT2 (txDAT-Cre) mouse line. Nested RT-PCR for β-actin, TH and Vglut2 from dissected VTA. Vglut2 wildtype band (500 bp) was observed in both txCtrl and txKO midbrain, while knockout (KO) band (KO; 250 bp) was only found in the gene targeted midbrain. β-actin and TH served as controls. **(C)** Number of TH positive neurons in the SN and VTA did not differ between txCtrl and txKO.Two-way ANOVA with Sidak *post hoc* for SN and VTA along three rostro-caudal section (txCtrl *n* = 3; txKO *n* = 3). **(D)** TH immunoreactivity in striatum and midbrain in txCtrl and txKO mice (scale bar 500 μm (striatum) and 50 μm (midbrain). **(E,F)** Cre-driven tdTom expression in ventral midbrain of eCtrl (**E**, top), eKO (**E**, bottom), txCtrl (**F**, top) and txKO (**F**, bottom) (scale bar 250 μm). Abbreviations: DStr, Dorsal striatum; NAcC, Nucleus accumbens core; NAcSh, Nucleus accumbens shell; SNc, Substantia nigra *pars compacta*; SNr, Substantia nigra pars reticulata; VTA, Ventral tegmental area; tdTom, tdTomato.

As presented above, we previously targeted the *Vglut2* gene in DA cells during development by breeding the eDAT-Cre transgenic mouse line (Ekstrand et al., 2007) with a floxed *Vglut2* allele (Wallén-Mackenzie et al., 2006) which generated the *Vglut2^eDAT-Cre^* KO mouse line (Birgner et al., 2010). Now, to direct VGLUT2 targeting to mature DA neurons, we bred the same *Vglut2* allele to the txDAT-Cre transgenic line, thus generating the new *Vglut2^txDAT-Cre^* inducible KO mouse line, in which *Vglut2* gene expression should be ablated by recombination upon tamoxifen treatment (Figure 4A). To verify recombination, nested RT-PCR analysis was performed on dissected VTA, which confirmed the presence of a Vglut2 KO band (smaller band as targeted *Vglut2* allele lacks exons 4–6) and a wildtype Vglut2 band in the *Vglut2^lx/lx;txDAT-Cre-tg^* (txKO) midbrain, while the control *Vglut2^lx/lx;txDAT-Cre-wt^* (txCtrl) midbrain only contained a wildtype Vglut2 band (Figure 4B). Having verified successful targeting of the *Vglut2* gene in adult mice using the txDAT-Cre-driver, immunohistochemistry for TH was used to assess the integrity of the DA system. No difference in histological appearance of TH immunofluorescence in the midbrain or in the striatal target areas was seen between txKO and txCtrl midbrain (Figures 4C,D). Last, we compared the appearance of the tdTom reporter in the VTA and SNc area between the previously published embryonal *Vglut2^eDAT-Cre^* transgenic line (Birgner et al., 2010; Alsiö et al., 2011) and the newly generated tamoxifen-induced *Vglut2^txDAT-Cre^* transgenic line. txCtrl and txKO midbrains of the *Vglut2^txDAT-Cre^* transgenic line were strikingly similar to each other (Figure 4E) and they were both similar to the eCtrl and eKO midbrains of the *Vglut2^eDAT-Cre^* transgenic mouse line (Figures 4E,F). Taken together, these results demonstrate that tamoxifen-induced activation of txDAT-Cre leads to successful ablation of *Vglut2* gene expression in the VTA and that this removal does not cause a prominent morphological phenotype in the mesostriatal DA system.

### Excitatory Postsynaptic Responses in Adult NAc Are Dependent on VGLUT2

**Figure 5:**
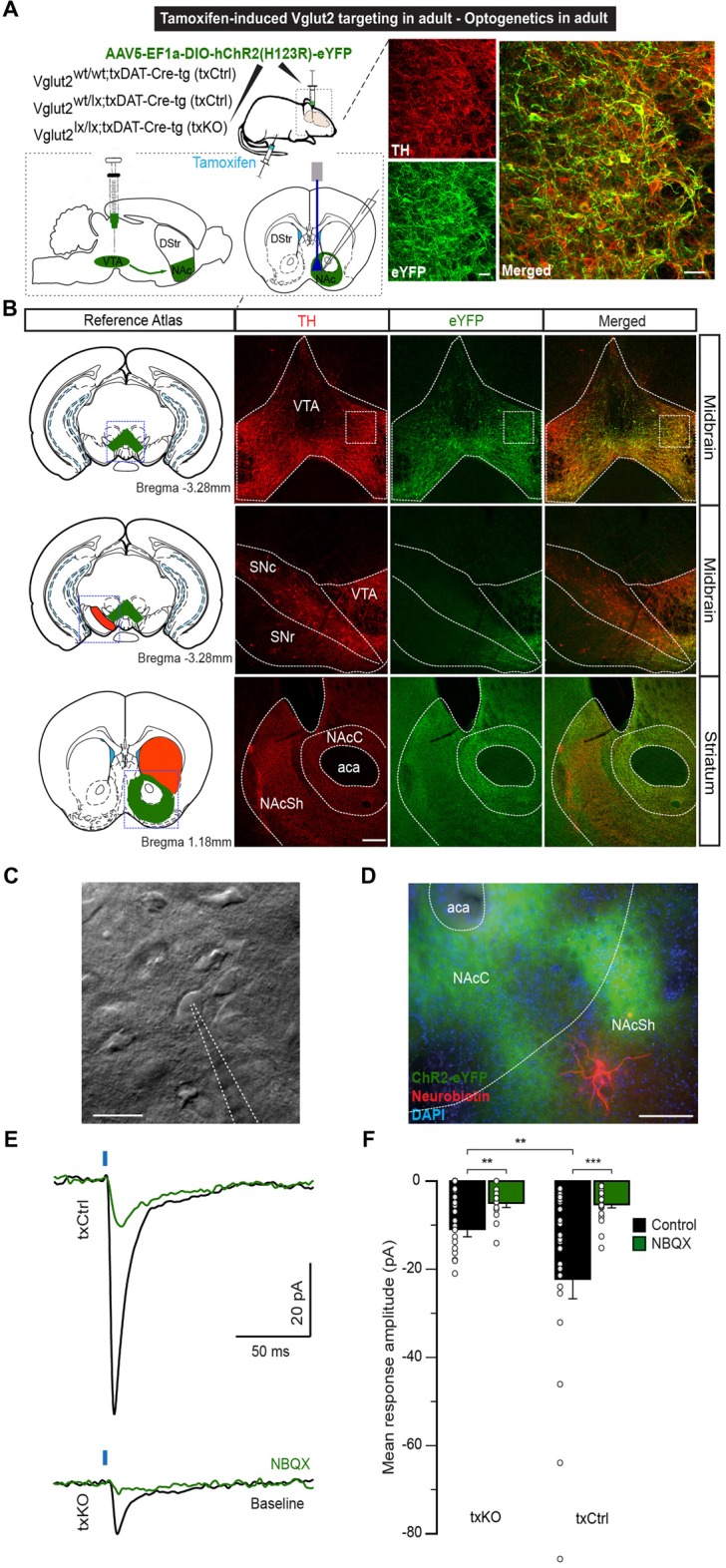
Optogenetics-driven stimulation of dopaminergic fibers in the NAcSh evokes VGLUT2-dependent responses. **(A)** Eight-week-oldtxCtrl and txKO mice were tamoxifen-treated and 1 week later stereotaxically injected with of AAV5-EF1a-DIO-hChR2(H123R)-eYFP into the VTA. Glutamate release was recorded in the NAcSh upon optical stimulation. **(B)** ChR2 expression was restricted to the VTA and colocalized with TH immunoreactivity. Inset: eYFP and TH immunofluorescence showing ample co-localization (scale bar 50 μm). No eYFP expression was detected in the SNc or SNr. ChR2 expression was detected in the projecting fibers to theNAc of the ventral striatum (scale bar 200 μm). **(C)** Representative example of accumbal cell imaged under IR-DIC and patched with 4–6 MΩ patch pipettes (scale bar 20 μm). **(D)** Patched neurons were filled with neurobiotin and stained with Cy5-conjugated streptavidin. Blue: DAPI, Green: eYFP, Red: Cy5 (scale bar 200 μm). **(E)** Representative average traces of light-induced responses in NAc neurons in txCtrl and txKO, before and after bath application of NBQX. **(F)** Light-evoked response amplitude for txKO and txCtrl mice. txKO exhibited smaller responses compared to txCtrl in control, but not under NBQX conditions. Unpaired Mann-Whitney test, **p < 0.01, ***p < 0.001 (txCtrl *n* = 25, 3 mice; txKO *n* = 24, 3 mice). Abbreviation: aca; anterior commissure, anterior part, DStr, Dorsal striatum; NAc, Nucleus accumbens; NAcC, Nucleus accumbens core; NAcSh, Nucleus accumbens shell; SNc, Substantia nigra pars compacta; SNr, Substantia nigra pars reticulata; VTA, Ventral tegmental area.

Using an optogenetic patch clamp approach, we and others have verified that embryonal targeting of VGLUT2 using the eDAT-Cre driver abolishes post-synaptic EPSCs in the NAc (Stuber et al., 2010; Hnasko et al., 2012; Wang et al., 2017). We now implemented similar methodology to confirm the loss of post-synaptic EPSCs in NAc upon txDAT-Cre-driven VGLUT2 targeting. One week after the last tamoxifen injection, *Vglut2^wt/wt;txDAT-Cre-tg^* or *Vglut2^wt/lx;txDAT-Cre-tg^* (txCtrl) and *Vglut2^lx/lx;txDAT-Cre-tg^* (txKO) mice were stereotaxically injected into the VTA with AAV5-EF1a-DIO-ChR2(H134)-eYFP for subsequent *ex vivo* slice patch clamp recordings of blue light-evoked responses in the NAc (Figure 5A). Histological analysis following the patch clamp recordings verified that ChR2-eYFP expression in cell bodies was restricted to the VTA and co-localized with TH immunoreactivity (Figures 5A,B). ChR2-eYFP expression was detected in mesoaccumbal projections and within the NAc (Figure 5B). During electrophysiological patch clamp recordings in NAc slice preparations (Figures 5C,D), light-evoked EPSCs with short onset latencies (2.8 ± 0.1 ms) were detected in most recorded neurons. Response amplitudes were significantly smaller in txKO mice compared to compared to txCtrl mice (Mann-Whitney U test, *p* = 0.005; Figures 5E,F). Bath application of AMPA antagonist 2,3-dihydroxy-6-nitro-7-sulfamoyl-benzo[f]quinoxaline-2,3-dione (NBQX) reduced light-evoked EPSCs by 71%, suggesting EPSCs were predominantly glutamatergic (Figure 5F and Supplementary Figures S2A,B). In some cases, we subsequently applied the NMDA antagonist (2R)-amino-5-phosphonovaleric acid (AP5) and observed a further decrease, but not complete abolishment, of light-evoked EPSCs (Supplementary Figure S2A). These experiments confirm the significant reduction of glutamatergic neurotransmission when *Vglut2* is gene-targeted in mature DA neurons.

### Amphetamine-Induced Locomotor Sensitization Unaffected by Tamoxifen-Induced VGLUT2 Targeting in Adulthood

**Figure 6:**
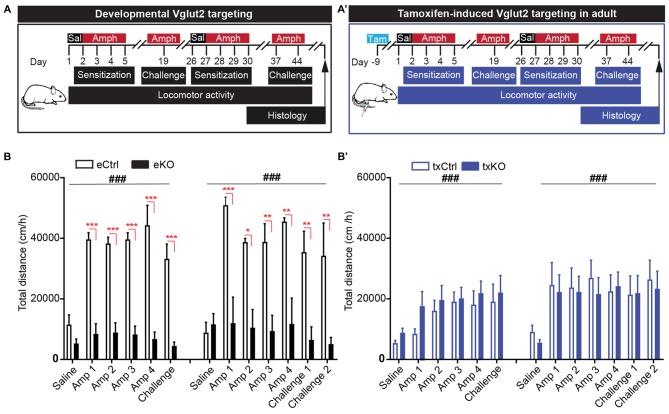
Amphetamine-induced locomotor sensitization is impeded upon VGLUT2 targeting from developing, but not mature, DA neurons. **(A,A′)** Schematic illustration of amphetamine-induced sensitization paradigm in **(A)** eCtrl and eKO or **(A′)** txCtrl and txKO mice. Animals received a single dose of saline (Day 1) followed by four consecutive injections of amphetamine (3 mg/kg; Days 2–5). Acute dose of amphetamine (Challenge) was administered after a washout period of 14 days. A week later (Day 26), the protocol was repeated and included two acute challenges (Challenge 1 and Challenge 2) on days 37 and day 44, respectively. **(B)** eKO show blunted response to amphetamine compared to eCtrl during both rounds of sensitization. There was no difference in locomotion between last days of repeated injection (Amp 4) and acute challenges. **(B′)** Both txCtrl and txKO showed an increase in amphetamine-induced locomotion compared to saline and no difference was observed between genotypes. Two-way repeated measures ANOVA with Sidak *post hoc* test (ANOVA ^###^*p* < 0.001; *post hoc* between genotype *p < 0.05, **p < 0.01, ***p < 0.001; eCtrl *n* = 3; eKO *n* = 3; txCtrl *n* = 7; txKO *n* = 11).

To address if the midbrain DA system has been functionally affected by the targeted deletion of VGLUT2 in mature DA neurons, an amphetamine-sensitization paradigm was implemented. In this experiment, we included both the embryonal *Vglut2^eDAT-Cre^* and the tamoxifen-induced *Vglut2^txDAT-Cre^* transgenic lines to enable a direct comparison between these mouse lines (Figure 6A,A′). All mice were given saline 1 day prior to initiation of four consecutive days of amphetamine injections. Upon 2 weeks of rest, the mice were given a challenge of one amphetamine dose (day 19). In accordance to our previous findings (Birgner et al., 2010), eKO mice showed a blunted response to amphetamine compared to eCtrl mice, that showed a potent psychostimulant-induced locomotion (two-way repeated measures ANOVA, treatment effect *F*_(5,20)_ = 9.87 *p* < 0.001, genotype effect *F*_(1,4)_ = 99.1 *p* = 0.001, interaction *F*_(5,20)_ = 9.79, *p* < 0.001). The degree of response did not differ between repeated or acute administration of amphetamine (Figure 6B). In contrast to the *Vglut2^eDAT-Cre^* transgenic line, loss of VGLUT2 from mature DA neurons did not seem to affect amphetamine-induced sensitization, as both txCtrl and txKO of the *Vglut2^txDAT-Cre^* transgenic line showed a similar increase in locomotion in response to amphetamine compared to saline (two-way repeated measures-ANOVA, treatment effect *F*_(5,80)_ = 4.99 *p* < 0.001, genotype effect *F*_(1,16)_ = 0.868 *p* = 0.37, interaction *F*_(5,80)_ = 0.32, *p* = 0.90; Figure 6B′).

To ascertain the behavioral response upon amphetamine sensitization further, we decided to perform a second round of amphetamine injections in the same mice. All mice therefore received amphetamine during four additional days (days 27–30), after which they were given one challenge 1 week (day 37) and then another challenge yet another week later (day 44; Figure 6A,A′). The results were similar to those seen during the first round of injections. There was a strong and significant difference between eCtrl and eKO mice, with eKO displaying a blunted response to amphetamine (eDAT-Cre two-way repeated measures ANOVA, treatment effect *F*_(6,24)_ = 6.64, *p* < 0.001, genotype effect *F*_(1,4)_ = 16.6 *p* = 0.02, interaction *F*_(6,24)_ = 6.33, *p* < 0.001). In contrast, no significant difference was seen between txCtrl and txKO (txDAT-Cre two-way repeated measures ANOVA treatment effect *F*_(6,96)_ = 6.62, *p* < 0.001, genotype effect *F*_(1,16)_ = 0.088 *p* = 0.77, interaction *F*_(6,96)_ = 0.25, *p* = 0.96), similarly to the first round of sensitization. The degree of locomotion did not differ between the first and last day of repeated amphetamine treatment or following acute injections of amphetamine on day 37 (challenge 1) and day 44 (challenge 2; Figure 6B,B′). These findings illustrate that DAT-Cre-driven targeting of *Vglut2* gene expression causes completely different behavioral effects in eKO (VGLUT2 targeting in embryogenesis) and txKO (VGLUT2 targeting in adulthood) mice, thus firmly demonstrating that the temporal onset of the VGLUT2 targeting event in DA neurons is crucial for behavioral outcome of relevance to drug addiction.

### Maintained Cocaine-Induced Locomotor Sensitization and Elevated Baseline AMPA/NMDA Ratio

**Figure 7:**
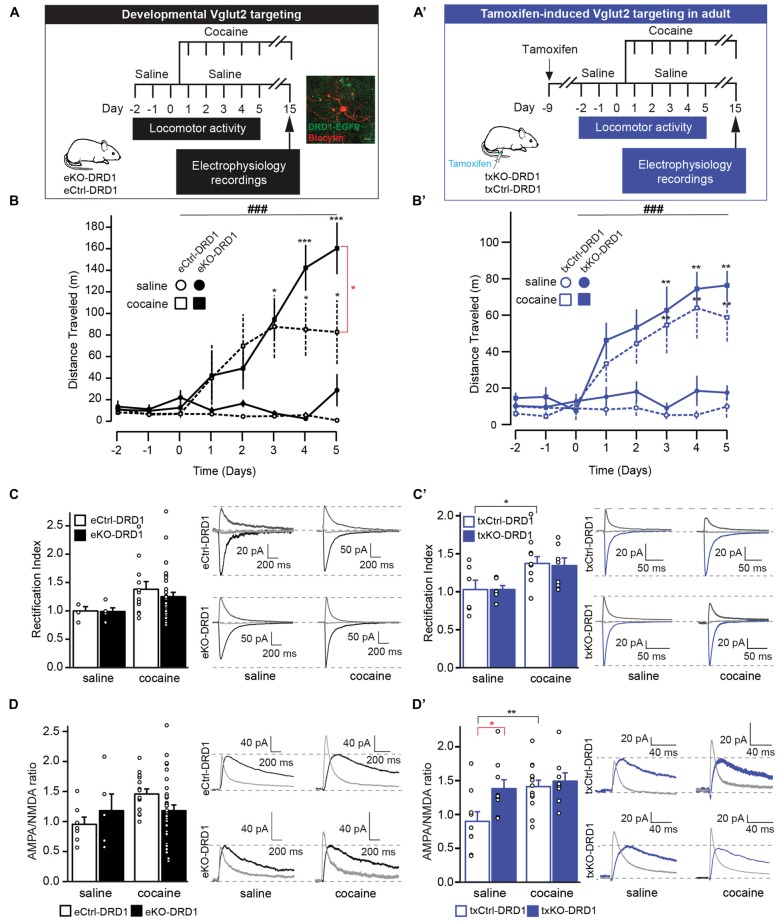
VGLUT2 targeting in mature, but not developing, DA results in maintained cocaine-induced sensitization and elevated baseline AMPA/NMDA ratio. **(A,A′)** Schematic timeline of cocaine-induced behavioral locomotor sensitization and subsequent electrophysiological recordings for **(A)** eKO-DRD1 and eCtrl-DRD1 and **(A′)** tamoxifen-induced txKO-DRD1 and txCtrl-DRD1 mice. All mice were habituated to the arena for 3 days during which they received saline injections (i.p.) Starting from “Day 1,” mice received either cocaine (20 mg/kg i.p.) or saline injections (i.p.) before behavioral testing for 5 days, then kept for additionally 10 days in their home cage. On Day 15, mice were sacrificed and whole-cell patch clamp experiments performed on brain slices. In **(A)** is also shown a fluorescent image of DRD1-expressing cells (EGFP; green) used in whole-cell patch clamp experiments filled with biocytin and stained with streptavidin (red). **(B,B′)** Cocaine-induced locomotor sensitization measured as distance travelled following saline or cocaine injections, respectively. **(B)** eKO-DRD1 and eCtr-DRD1. **(B′)** txKO-DRD1 and txCtrl-DRD1. **(C)** Rectification index (RI) and raw traces recorded cells from eKO-DRD1 and eCtrl-DRD1 mice treated with saline or cocaine at −70 mV (black), 0 mV (light gray) and +40 mV (dark gray). **(C′)** RI and raw traces recorded cells from txKO-DRD1 and txCtrl-DRD1 mice treated with saline or cocaine at −70 mV (blue), 0 mV (light gray) and +40 mV (dark gray). **(D)** AMPA/NMDA ratio and raw traces of cells from eKO-DRD1 and eCtrl-DRD1 mice treated with saline or cocaine for NMDA current (black); AMPA current (light gray). **(D′)** AMPA/NMDA ratio and raw traces of cells from txKO-DRD1 and txCtrl-DRD1 mice treated with saline or cocaine for NMDA current (blue); AMPA current (light gray). Two-way repeated measures ANOVA Tukey’s *post hoc* (behavior) and two-way ANOVA Sidak *post hoc* (electrophysiology) (ANOVA ^###^*p* < 0.001; *post hoc* between genotype *p < 0.05 and *post hoc* between treatment of same genotype: *p < 0.05, **p < 0.01, ***p < 0.001; Behavior: saline: eCtrl, *n* = 3; eKO, *n* = 2, cocaine: eCtrl, *n* = 5; eKO, *n* = 5, saline: txCtrl, *n* = 4; txKO, *n* = 4, cocaine: txCtrl, *n* = 9; txKO, *n* = 8; electrophysiology: RI saline: eCtrl, *n* = 4, eKO, *n* = 5; cocaine: eCtrl, *n* = 12,: eKO, *n* = 28; saline txCtrl, *n* = 6, txKO, *n* = 6; cocaine txCtrl, *n* = 12: txKO, *n* = 7; AMPA/NMDA ratio saline: eCtrl, *n* = 7, eKO, *n* = 5; cocaine: eCtrl, *n* = 15; eKO, *n* = 33; saline: txCtrl, *n* = 9, txKO, *n* = 9; cocaine: txCtrl, *n* = 13, tKO, *n* = 7). All mice expressed the DRD1-EGFP transgene.

Repeated exposure to cocaine causes lasting adaptations of excitatory synaptic transmission onto DRD1-MSNs in the NAc (Engblom et al., 2008; Mameli et al., 2009; Pascoli et al., 2011, 2014). With repeated cocaine exposure, calcium-permeable AMPA receptors are inserted into accumbal DRD1-MSNs, which is reflected by an increase in the AMPA to NMDA ratio and rectifying AMPAR-mediated currents in these cells. The prefrontal cortex, amygdala and hippocampus provide glutamatergic input to these neurons and mediate behavioral features of drug-adaptive behavior, including locomotor sensitization (Creed et al., 2015; Pascoli et al., 2015). The putative contribution of glutamate co-release from glutamate-DA neurons in the VTA to alterations in cocaine-evoked plasticity in DRD1-MSNs and subsequent behavior has never been investigated. To enable the analysis of putative contribution from VGLUT2-mediated co-release during development and in adulthood, we included both the embryonal *Vglut2^eDAT-Cre^* and the tamoxifen-induced *Vglut2^txDAT-Cre^* transgenic lines. To identify DRD1-MSNs in accumbal slice preparations and to enable the comparison between developmental and adult VGLUT2 targeting, both the *Vglut2^eDAT-Cre^* and the *Vglut2^txDAT-Cre^* mouse lines were bred with a DRD1-EGFP reporter line generating eCtrl, eKO, txCtrl and txKO mice expressing the DRD1-EGFP reporter (referred to as eCtrl-DRD1, eKO-DRD1, txCtrl-DRD1, txKO-DRD1). Mice were subjected to a cocaine-induced sensitization paradigm and following 10 days of cocaine withdrawal, patch clamp recordings were performed in DRD1-MSNs of the NAc (Figure 7A,A′). Cocaine administration resulted in increased locomotion with significantly higher activity compared to saline seen already on day 3 in both mouse lines irrespective of genotype (eDAT-Cre two-way repeated measures ANOVA Effect of, time effect *F*_(7,77)_ = 7.43, *p* < 0.001, treatment effect *F*_(3,11)_ = 3.93, *p* = 0.04, interaction *F*_(21,77)_ = 3.81, *p* < 0.001; txDAT-Cre two-way repeated measures ANOVA, time effect *F*_(7,147)_ = 10.1, *p* < 0.001, treatment effect *F*_(3,21)_ = 5.59, *p* = 0.006, interaction *F*_(21,147)_ = 3.17, *p* < 0.001; Figure 7B,B′). However, whilst both eKO-DRD1 and txKO-DRD1 covered greater distance compared to their equivalent controls following cocaine treatment, a significant difference between genotypes was only detected on day 5 between eKO-DRD1 and eCtrl-DRD1 (Tukey’s *post hoc* *p* = 0.01) and not between txKO-DRD1 and txCtrl-DRD1 (Tukey’s *post hoc* *p* = 0.47; Figure 7B,B′).

Electrically-evoked EPSCs from DRD1-MSNs were subsequently recorded at different holding potentials (−70 mV, 0 mV, +40 mV) under bath-application of 100 μm picrotoxin and 50 μm APV to isolate AMPA currents. Whole-cell patch clamp recordings *ex vivo* did not reveal differences in inward rectification between treatment nor genotype (two-way ANOVA, treatment effect *F*_(1,45)_ = 2.25, *p* = 0.14; genotype effect *F*_(1,45)_ = 0.03, *p* = 0.86, interaction *F*_(1,45)_ = 0.02, *p* = 0.90) with RI of 1.00 ± 0.08 for eCtrl-DRD1 and 0.99 ± 0.07 for eKO-DRD1 under baseline conditions and 1.39 ± 0.14 for eCtrl-DRD1 and 1.32 ± 0.14 for eKO-DRD1 following cocaine (Figure 7C with representative trace). In contrast, patched cells from the inducible DAT-Cre mouse line did not differ between the genotypes after saline administration but showed an inward rectification following cocaine administration for the txCtrl-DRD1 compared to saline with rectification indices (RI) of 1.03 ± 0.13 for txCtrl-DRD1 and 1.03 ± 0.06 for txKO-DRD1 under saline, 1.37 ± 0.09 for txCtrl-DRD1 1.34 ± 0.11 for txKO-DRD1 with cocaine (two-way ANOVA treatment effect *F*_(1,27)_ = 10.1, *p* = 0.004, genotype effect *F*_(1,27)_ = 0.02, *p* = 0.90, interaction *F*_(1,27)_ = 0.02, *p* = 0.89; Sidak *post hoc* saline vs. cocaine txCtrl *p* = 0.04; txKO *p* = 0.10; Figure 7C′ with representative trace).

The NMDA current was obtained by subtraction of evoked current at +40 mV before and after the application of 50 μm of D-AP5. Targeted deletion of VGLUT2 from DA neurons during development did not affect the AMPA/NMDA ratio under saline or cocaine conditions between or within genotype with ratios of 0.96 ± 0.12 and 1.19 ± 0.28 for eCtrls-DRD1 and eKO-DRD1, respectively, under saline and ratios of 1.47 ± 0.08 (eCtrl-DRD1) and 1.19 ± 0.09 (eKO-DRD1) under cocaine (two-way ANOVA treatment effect *F*_(1,56)_ = 2.59, *p* = 0.11, genotype effect *F*_(1,56)_ = 0.03, *p* = 0.87, interaction *F*_(1,56)_ = 2.58, *p* = 0.11 (Figure 7D). A significant effect of cocaine treatment and genotype was, however, detected in the inducible line (two-way ANOVA treatment effect *F*_(1,35)_ = 6.28, *p* = 0.017, genotype effect *F*_(1,35)_ = 5.08, *p* = 0.03, interaction *F*_(1,35)_ = 2.57, *p* = 0.118). A significant higher AMPA/NMDA ratio was observed in saline-treated txKO-DRD1 mice (1.38 ± 0.13) with respect to saline-treated txCtrls-DRD1 (0.9 ± 0.15; Sidak *post hoc* *p* = 0.023). As expected cocaine treatment significantly increased the AMPA/NMDA ratio in txCtrl-DRD1 mice (Sidak *post hoc* *p* = 0.008; Figure 7D′). Interestingly, and in contrast to the txCtrl-DRD1 mice, the elevated AMPA/NMDA ratio detected at baseline conditions in txKO-DRD1 mice prevented cocaine treatment to lead to significantly higher AMPA/NMDA ratio in those mice (Sidak *post hoc* *p* = 0.80; Figure 7D′). Finally, no difference was detected between cocaine-treated txCtrl-DRD1 and txKO-DRD1 mice with ratios of 1.41 ± 0.09 and 1.49 ± 0.13, respectively (Sidak *post hoc* *p* = 0.871; Figure 7D′). In summary, while no significant effects were observed upon VGLUT2 targeting in embryogenesis, removal of VGLUT2 in mature DA neurons seems sufficient to increase AMPA/NMDA ratio in accumbal MSNs to such a level that cocaine-evoked plasticity is occluded further. This experiment reveals that VGLUT2-mediated glutamate co-release from mature VTA DA neurons is crucial for maintaining baseline AMPA/NMDA ratio of MSNs in the mesoaccumbal projection.

## Discussion

In this study, we created a tamoxifen-inducible *Vglut2^txDAT-Cre^* mouse line to selectively probe the role of VGLUT2 in mature DA neurons. We show that this temporally restricted *Vglut2* gene ablation in DAT-Cre-expressing DA neurons causes a significant decrease in glutamatergic neurotransmission in the NAc, thus demonstrating that mesoaccumbal glutamate release from VTA DA neurons is mediated via VGLUT2. The main behavioral findings we present are that *Vglut2^txDAT-Cre^* txKO mice showed the same elevated level of locomotor response upon injections of the addictive substances amphetamine and cocaine as observed in control animals, a finding which is strikingly different from the blunted phenotype observed in *Vglut2^eDAT-Cre^* eKO mice, in which VGLUT2 is deleted from embryogenesis. Finally, we demonstrate that glutamate release from mature VTA DA neurons significantly affects synaptic plasticity of MSNs in the NAc. By measuring the AMPA/NMDA ratio of DRD1-expressing MSNs, we identified a strongly elevated baseline AMPA/NMDA ratio in txKO compared to control mice. This effect was sufficiently potent to occlude any further effect on this measure of synaptic plasticity by cocaine. It is known that MSNs receive glutamatergic terminals from forebrain neurons onto the head of the dendritic spine, while VTA-derived DA reaches the neck region and can modulate the responsiveness of MSN spines to excitatory transmission (Russo et al., 2010; Yetnikoff et al., 2014). This interaction between midbrain dopaminergic and forebrain glutamatergic neurons has been established as a core feature for several stages of addiction, including drug-seeking and relapse (Lüscher and Malenka, 2011; Pascoli et al., 2015). The current results show for the first time that VGLUT2-mediated glutamatergic transmission from mature VTA DA neurons contributes to this critical interaction on NAc MSNs.

The role of VGLUT2 in mature DA neurons has been a matter of debate not least since the use of different experimental approaches in different laboratories has made it difficult to establish the amount of VTA DA neurons that express this transporter (El Mestikawy et al., 2011; Trudeau et al., 2014). VTA DA neurons develop in the embryonal midbrain from midgestation with TH immunoreactivity appearing in post-mitotic neurons already around E11 followed by DAT at E13 (Ang, 2006). Vglut2 mRNA has been observed within a subset of post-mitotic TH-positive DA neurons as early as E12.5 (Birgner et al., 2010; Nordenankar et al., 2014). Using fluorescent *in situ* hybridization for Vglut2, Th and Dat mRNAs, we now show that the mouse *Vglut2* gene has a peak of co-expression with endogenous Th and Dat around birth, with only sparse co-expression detected in embryonal (E14.5) and adult DA neurons. Notably, as thoroughly described in the rat (reviewed in Morales and Margolis, 2017), and confirmed in the present study, Vglut2 mRNA is stronger in the medial than lateral aspect of the VTA. However, Dat mRNA, but not Th mRNA, shows the opposite expression pattern with stronger lateral than medial expression. These inverse expression patterns between *Vglut2* and *Dat* genes help explain the current observation that the extent of co-localization between Vglut2 and Th mRNAs is higher than between Vglut2 and Dat mRNAs. The histological spatio-temporal mapping of Vglut2, Th and Dat mRNA performed in the present study should be useful to increase the understanding of the glutamate-DA co-releasing cells and their role in mesoaccumbal neurocircuitry.

It is noteworthy that not all midbrain DA neurons co-express the *Th* and *Dat* genes. This is of particular interest in the transgenic context when using their promoters to drive Cre-recombinase for gene targeting of floxed alleles (Lammel et al., 2015; Pupe and Wallén-Mackenzie, 2015). For example, expression of the *Vglut2* gene in Th-positive cells lacking *Dat* gene expression is of interest since not only different DAT-Cre-drivers (Birgner et al., 2010; Hnasko et al., 2010; Alsiö et al., 2011; Fortin et al., 2012), but also a TH-Cre-driver has been used to target *Vglut2* gene expression in mice (Nordenankar et al., 2014). This targeting event resulted in somewhat different phenotypes than obtained upon DAT-Cre-mediated targeting and based on an observed promiscuity of the TH-Cre driver in non-monoaminergic cells, the obtained phenotype was difficult to pinpoint (Nordenankar et al., 2014).

Despite the low amount of VTA DAT neurons containing Vglut2 mRNA observed in the histological analyses of the adult mouse midbrain, our electrophysiological data corroborate previous findings showing that optogenetic stimulation of mature DAT-Cre neurons in the VTA causes EPSCs in MSNs of the NAc (Stuber et al., 2010; Tecuapetla et al., 2010; Mingote et al., 2015). We can show that these currents significantly decrease in amplitude upon VGLUT2 ablation in mature VTA DA neurons. This finding is similar, but not identical, to previous analyses where optogenetic stimulations were performed in the embryonal *Vglut2^eDAT-Cre^* eKO mice, in which glutamate co-release was found completely abolished (Hnasko et al., 2010; Stuber et al., 2010; Wang et al., 2017). The results we present in the current study using tamoxifen-induced VGLUT2 targeting in adulthood demonstrate that glutamate co-release in mature DA neurons is to a large part dependent on VGLUT2.

When comparing previous reports of developmental *Vglut2^eDAT-Cre^* targeting (Birgner et al., 2010; Hnasko et al., 2010; Alsiö et al., 2011; Fortin et al., 2012) with the phenotypes obtained in the new *Vglut2^txDAT-Cre^* targeting of VGLUT2 in mature DA neurons presented here, a major difference is the locomotor response to psychostimulants. By comparing *Vglut2^eDAT-Cre^* eKO and *Vglut2^txDAT-Cre^* txKO and relevant control mice within the same experimental sensitization setup, we can firmly demonstrate that adult VGLUT2 targeting does not affect amphetamine-induced locomotor response while, as previously shown (Birgner et al., 2010), developmental targeting causes a strongly blunted response. This observation suggests that down-regulation of *Vglut2* gene expression levels from embryogenesis might pre-dispose to addictive-like behavior. Indeed, in a study of common haplotype tag-single nucleotide polymorphism, SNP, in the VGLUT genes in individuals suffering from substance use disorder, one SNP of the VGLUT2 gene (rs2290045) showed significant association with severe alcoholism (Comasco et al., 2014).

Synaptic plasticity, such as long-term potentiation and long-term depression (Malinow and Malenka, 2002; Kessels and Malinow, 2009) is also implicated in behavioral sensitization, in which repeated exposure to cocaine results in an increased locomotor response (Thomas et al., 2001). Cocaine administration has been shown to result in an increase of AMPA-mediated currents in DRD1-MSNs of the NAc (Pascoli et al., 2011; Creed et al., 2015) and replacement of GluR2-containing AMPA receptors to ones lacking the GluR2 subunit in VTA DA neurons (Bellone and Lüscher, 2006; Mameli et al., 2007). GluR2-lacking AMPA receptors exhibit higher peak conductance, permeability for Ca^2+^ and, as a result, inward-rectifying properties (Conrad et al., 2008; Wolf and Ferrario, 2010). In the present study, DRD1-MSNs in the NAc of *Vglut2^txDAT-Cre^* txKO mice exhibited a higher AMPA/NMDA ratio than txCtrl mice at saline conditions, which was, however, not matched by inward-rectification, indicating the maintained presence of GluR2-containing AMPA receptors in txKO under baseline conditions. Following cocaine administration, DRD1-MSN of both txKO and txCtrl mice exhibited an increase in RI. The shift coincided with an increase in AMPA/NMDA ratio in the txCtrl, while the AMPA/NMDA ratio was not further altered in *Vglut2^txDAT-Cre^* txKO animals, suggesting that the synapses in the txKO are already potentiated due to disrupted glutamate co-release. This enhanced baseline level of AMPA/NMDA ratio may mask cocaine-induced plasticity as seen in txCtrl, and in turn explain the absent differences between the two genotypes in cocaine-induced behavioral sensitization. Based on these new observations, we propose that disruption of glutamate co-release in mature dopaminergic neurons of the VTA leads to alterations in baseline AMPA and NMDA currents in DRD1-MSN.

Taken together with our observations of significantly reduced glutamatergic post-synaptic currents in the MSN, our study shows that ablation of VGLUT2 in the mature mesoaccumbal DA circuitry leads to a measurable effect on both glutamatergic neurotransmission and synaptic plasticity of MSNs in the NAc. Since no study has addressed the consequence of VGLUT2-mediated glutamate co-release on AMPA/NMDA ratio in accumbal MSNs before, further studies will be required to fully explain the mechanism on molecular level. Clearly, ablation of VGLUT2 in adulthood followed through by behavioral and electrophysiological measurements within a restricted time span has the added benefit of a substantially restricted level of compensatory adaptations that might occur when ablation takes place during embryonal brain development. Indeed, we have previously shown that eKO mice have higher baseline levels of immediate early genes *c-fos* and *Nur77* in the striatal complex, to such a level that cocaine sensitization failed to increase them further (Alsiö et al., 2011). The current txKO line should be of particular use in the context of co-release in behavioral and molecular plasticity.

In this study, we pin-point that ablation of VGLUT2-mediated co-release from midbrain DA neurons during adulthood has a significant effect on AMPA/NMDA ratio but leaves amphetamine- and cocaine induced locomotion intact. While *Vglut2^txDAT-Cre^* txKO mice show normal locomotor sensitization in the cocaine paradigm, we had expected, based on previous studies (Hnasko et al., 2010), a blunted cocaine-induced locomotor response in the *Vglut2^eDAT-Cre^* eKO mice. However, in this experiment, there was no overall difference between genotypes in the *Vglut2^eDAT-Cre^* line, apart from the last day of cocaine administration in which eKO mice had higher locomotion than controls. This discrepancy is likely due to different genetic backgrounds between mice used in different laboratories, which highlights a need for common animal pools to enable direct comparison in terms of behavior and circuitry function. Together with our current observation, these findings support the need for genetic models implementing targeting in adulthood when attempting to study effects of manipulated glutamatergic neurotransmission. In addition, a recent study has demonstrated circadian-dependance in DA-related behavior influenced by VGLUT3, a sister molecule of VGLUT2 which is present in striatal cholinergic interneurons (Divito et al., 2015), showing that also wake-sleep cycle should be considered when addressing the role of VGLUTs in behavior, and also when comparing data obtained in different laboratories. Further, in contrast to targeting at a distinct time-point during adulthood, developmental targeting of VGLUT2 in DA neurons likely causes compensatory adaptations which may help explain the lack of post-synaptic phenotype in recorded MSNs. However, the relatively low amount of recording points might also contribute to the lack of statistical significance in this experiment. Further analyses would be crucial in order to fully understand the post-synaptic circuitry effects by glutamate-DA co-release.

Despite first described some two decades ago, the lack of selective experimental approaches has made it difficult to clarify the putative role of glutamate co-release from midbrain DA neurons in behavioral reinforcement and addiction (Pupe and Wallén-Mackenzie, 2015; Morales and Margolis, 2017). The current implementation of temporally controlled targeting in adulthood advances the knowledge of VGLUT2 in DA neurons and complements previous gene targeting studies performed in the developing mouse brain. To summarize, in the absence of VGLUT2 in mature DA neurons at adulthood, mice show normal psychostimulant-induced locomotion, while on a molecular level, the loss of VGLUT2 causes a significant decrease of mesoaccumbal glutamatergic neurotransmission and an elevated baseline AMPA/NMDA ratio, sufficiently high to occlude any further effect on neurotransmission by the addictive drug cocaine. Together with previously published data, our present results point towards a dual role for *Vglut2* gene expression within midbrain DA neurons. During development, VGLUT2 is essential for establishing and maintaining the mesoaccumbal DA system, as its loss causes severe dysfunction in DA release and abnormal behavioral response to addictive substances as well as sugar (Birgner et al., 2010; Hnasko et al., 2010; Alsiö et al., 2011; Fortin et al., 2012). While *Vglut2* expression levels in the *Dat-*expressing population of VTA DA neurons are high around birth, we show that there is a substantial down-regulation of Vglut2 mRNA in mature DA neurons. However, VGLUT2 in VTA DA neurons is still crucial in adulthood for maintaining mesoaccumbal glutamate transmission as well as synaptic strength in accumbal MSNs. Synaptic plasticity has never been studied in the context of mesoaccumbal glutamate co-release before, but the current study highlights VGLUT2 as an emerging player in the complex mechanisms of substance addiction that should be investigated further.

## Author Contributions

MP planned experiments, performed histological experiments, analyzed data and wrote the manuscript. MC performed cocaine experiments and analyzed data. MD performed glutamate recordings and analyzed data. ZB performed stereotaxic injections and analyzed data. SD performed *in situ* hybridization experiments and analyzed data. HP performed amphetamine sensitization experiments. CB performed cocaine experiments and analyzed data. GS supervised glutamate recordings. CL supervised cocaine experiments. ÅW-M conceived and planned all experiments, analyzed data and wrote the manuscript. All authors reviewed the manuscript.

## Conflict of Interest Statement

SD is the owner of Oramacell. The remaining authors declare that the research was conducted in the absence of any commercial or financial relationships that could be construed as a potential conflict of interest. The handling Editor declared a shared affiliation, though no other collaboration, with several of the authors MP, ZB, HP, ÅW-M.
