# Supplementary figures and images for: Targeting VGLUT2 in Mature Dopamine Neurons Decreases Mesoaccumbal Glutamatergic Transmission and Identifies a Role for Glutamate Co-release in Synaptic Plasticity by Increasing Baseline AMPA/NMDA Ratio
Source: Front Neural Circuits. 2018 Aug 29;12:64. doi: 10.3389/fncir.2018.00064 (PMC6123381; doi:10.3389/fncir.2018.00064)

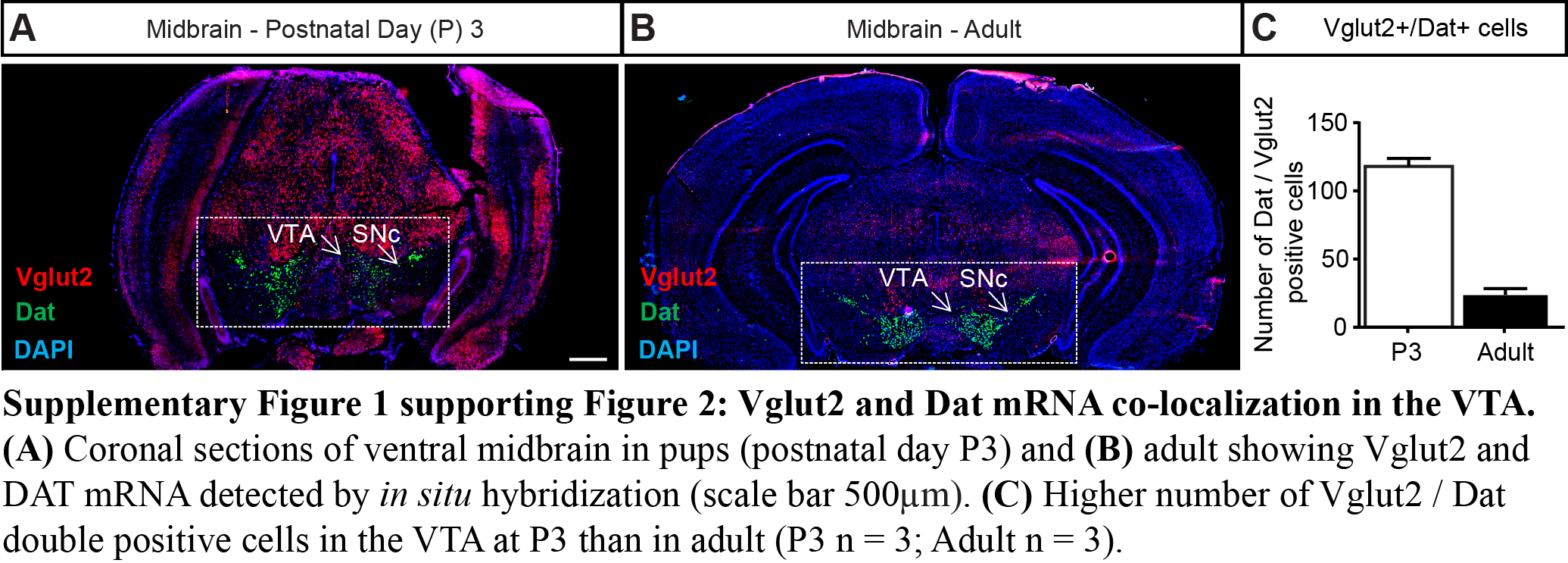

Supplement: Supplementary file 1 [file Image_1.jpg]

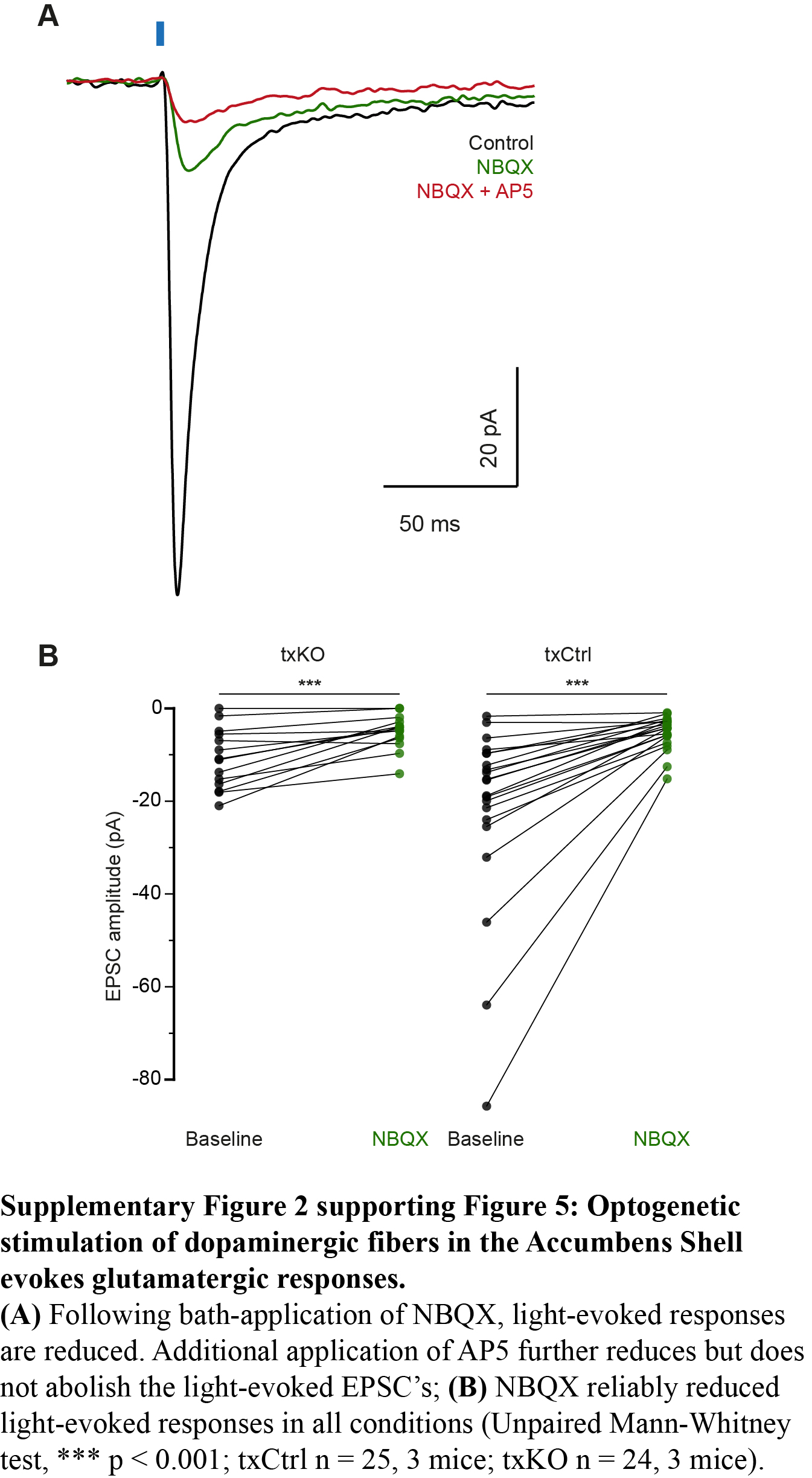

Supplement: Supplementary file 2 [file Image_2.JPEG]
