# Supplementary material for: Targeting VGLUT2 in Mature Dopamine Neurons Decreases Mesoaccumbal Glutamatergic Transmission and Identifies a Role for Glutamate Co-release in Synaptic Plasticity by Increasing Baseline AMPA/NMDA Ratio
Source: Front Neural Circuits. 2018 Aug 29;12:64. doi: 10.3389/fncir.2018.00064 (PMC6123381; doi:10.3389/fncir.2018.00064)
Supplement: Supplementary file 3 [file Table_1.DOCX]

Supplementary Material

# Targeting VGLUT2 in mature dopamine neurons decreases mesoaccumbal glutamatergic transmission and identifies a role for glutamate co-release in synaptic plasticity by increasing baseline AMPA/NMDA ratio

**Maria Papathanou^1^,** Meaghan Creed^2#^, Matthijs Dorst^3^, Zisis Bimpisidis^1^, Hanna Pettersson^1^, Sylvie Dumas^4^, Camilla Bellone^2^, Gilad Silberberg^3^, Christian Lüscher^2,5^, **Åsa Wallén-Mackenzie^1,*^**

*** Correspondence: Åsa Wallén-Mackenzie**: [asa.mackenzie@ebc.uu.se](mailto:asa.mackenzie@ebc.uu.se)

# Supplementary Table 1: PCR primer sequences used for genotyping of transgenic mice used in the study.

| **Transgene** | **Direction** | **PCR primer sequence** |
| --- | --- | --- |
| Dat-Cre | fw | 5'-ACGAGTGATGAGGTTCGCAAGA-3' |
| Dat-Cre | rev | 5'-ACCGACGATGAAGCATGTTTAG-3' |
| TdTomato | fw (mut) | 5'-CTGTTCCTGTACGGCATGG-3' |
| TdTomato | rev (mut) | 5'-GGCATTAAAGCAGCGTATCC-3' |
| TdTomato | fw (wt) | 5'-AAGGGAGCTGCAGTGGAGTA-3' |
| TdTomato | rev (wt) | 5'-CCGAAAATCTGTGGGAAGTC-3' |
| DRD1-eGFP | fw | 5'-ACCGGAAGTGCTTTCCTTCTGGA-3' |
| DRD1-eGFP | rev | 5'-TAGCGGCTGAAGCACTGCA-3' |
| Vglut2-Lox | fw | 5'-CAGGCAAAATCTGTCCACCT-3' |
| Vglut2-Lox | rev | 5'-AGGGTAGGCCAAAAGCAATC-3' |
| DATCreERT2 | fw | 5'-GGCTGGTGTGTCCATCCCTGAA-3' |
| DATCreERT2 | rev | 5'-GGTCAAATCCACAAAGCCTGGCA-3' |
