# Supplementary material for: Targeting VGLUT2 in Mature Dopamine Neurons Decreases Mesoaccumbal Glutamatergic Transmission and Identifies a Role for Glutamate Co-release in Synaptic Plasticity by Increasing Baseline AMPA/NMDA Ratio
Source: Front Neural Circuits. 2018 Aug 29;12:64. doi: 10.3389/fncir.2018.00064 (PMC6123381; doi:10.3389/fncir.2018.00064)
Supplement: Supplementary file 4 [file Table_2.DOCX]

Supplementary Material

# Targeting VGLUT2 in mature dopamine neurons decreases mesoaccumbal glutamatergic transmission and identifies a role for glutamate co-release in synaptic plasticity by increasing baseline AMPA/NMDA ratio

**Maria Papathanou^1^,** Meaghan Creed^2#^, Matthijs Dorst^3^, Zisis Bimpisidis^1^, Hanna Pettersson^1^, Sylvie Dumas^4^, Camilla Bellone^2^, Gilad Silberberg^3^, Christian Lüscher^2,5^, **Åsa Wallén-Mackenzie^1,*^**

*** Correspondence: Åsa Wallén-Mackenzie**: [asa.mackenzie@ebc.uu.se](mailto:asa.mackenzie@ebc.uu.se)

# Supplementary Table 2: Primer sequences and thermal conditions for nested PCR used for verifying txKO mice

Primers for PCR 1

| **Transgene** | **Direction** | **PCR primer sequence** |
| --- | --- | --- |
| β-actin | Fw | 5'-CTCTTTTCCAGCCTTCCTTCTT-3' |
| β-actin | Rev | 5'-AGTAATCTCCTTCTGCATCCTGTC-3' |
| TH | NL-Fw | 5'-GTTCTCAACCTGCTCTTCTCCTT-3' |
| TH | NL-Rev | 5'-GGTAGCAATTTCCTCCTTTGTGT-3' |
| Vglut2 | NL-Fw | 5'-GCCGCTACATCATAGCCATC-3' |
| Vglut2 | NL-Rev | 5'-GCTCTCTCCAATGCTCTCCTC-3' |

Primers for PCR 2

| **Transgene** | **Direction** | **PCR primer sequence** |
| --- | --- | --- |
| β-actin | Fw | 5'-CTCTTTTCCAGCCTTCCTTCTT-3' |
| β-actin | Rev | 5'-AGTAATCTCCTTCTGCATCCTGTC-3' |
| TH | Fw | 5'-GTACAAAACCCTCCTCACTGTCTC-3' |
| TH | Rev | 5'-CTTGTATTGGAAGGCAATCTCTG-3' |
| Vglut2 | Fw | 5'-ACATGGTCAACAACAGCACTATC-3' |
| Vglut2 | Rev | 5'-ATAAGACACCAGAAGCCAGAACA-3' |

Thermal conditions of PCR1

| **Temperature** | **Time** | **Cycles** |
| --- | --- | --- |
| 98° C | 30 s | 1’ |
| 98° C  53° C  72° C | 10 s  30 s  15 s | 20’ |
| 72° C | 5 min | 1’ |

Thermal conditions of PCR2

| **Temperature** | **Time** | **Cycles** |
| --- | --- | --- |
| 98° C | 30 s | 1’ |
| 98° C  53° C  72° C | 10 s  30 s  15 s | 30’ |
| 72° C | 5 min | 1’ |
